# A Late Holocene community burial area: Evidence of diverse mortuary practices in the Western Cape, South Africa

**DOI:** 10.1371/journal.pone.0230391

**Published:** 2020-04-16

**Authors:** Susan Pfeiffer, Judith Sealy, Lesley Harrington, Emma Loftus, Tim Maggs

**Affiliations:** 1 Department of Anthropology, University of Toronto, Toronto, Ontario, Canada; 2 Department of Archaeology, University of Cape Town, Cape Town, Western Cape, South Africa; 3 Center for the Advanced Study of Human Paleobiology, George Washington University, Washington, DC, United States of America; 4 Department of Anthropology, University of Alberta, Edmonton, Alberta, Canada; 5 McDonald Institute for Archaeological Research, University of Cambridge, Cambridge, United Kingdom; University of Otago, NEW ZEALAND

## Abstract

Over several decades, human skeletal remains from at least twelve individuals (males, females, children and infants) were recovered from a small area (ca. 10 x 10 m) on the eastern shore of Table Bay, Cape Town, near the mouth of the Diep River where it empties into the sea. Two groups, each comprising four individuals, appear to have been buried in single graves. Unusually for this region, several skeletons were interred with large numbers of ostrich eggshell (OES) beads. In some cases, careful excavation enabled recovery of segments of beadwork. One collective burial held items including an ostrich egg-shell flask, a tortoise carapace bowl, a fragmentary bone point or linkshaft and various lithic artefacts. This group appears to have died together and been buried expediently. A mid-adult woman from this group sustained perimortem blunt-force trauma to her skull, very likely the cause of her death. This case adds to the developing picture of interpersonal violence associated with a period of subsistence intensification among late Holocene foragers. Radiocarbon dates obtained for nine skeletons may overlap but given the uncertainties associated with marine carbon input, we cannot constrain the date range more tightly than 1900–1340 calBP (at 2 sigma). The locale appears to have been used by a community as a burial ground, perhaps regularly for several generations, or on a single catastrophic occasion, or some combination thereof. The evidence documents regional and temporal variation in burial practices among late Holocene foragers of the south-western Cape.

## Introduction

The mortuary practices of past communities can reflect the contexts of members’ deaths. They may also reflect the broader circumstances of the communities themselves. Mortuary places are products of place-making activities. For hunter-gatherers, these places may provide signals about land tenure and about social identity [1:Fig 4.21]. Archaeological interpretations of death and burial have historically been built on ethnographic analogy [2]. As framed in what has been called the Saxe-Binford program [3], ethnographic analogy predicts that grave goods can be interpreted as evidence of the differential status of the deceased, and the establishment of a cemetery implies the existence of territoriality. Immediate-return forager communities [4] like the northern KhoeSan people of southern Africa, maintain fundamentally egalitarian social systems, the principles of which may be in conflict with a high degree of differential personal status. Territoriality among northern KhoeSan groups existed through mechanisms of social boundary defense, with groups using approaches that ranged from hospitality to hostility [5].

In southern Africa, socio-economies that lack evidence for herding and that occur between about 4,000 and 100 years ago are associated with the final Later Stone Age (LSA) [see 6 for the South African cultural sequence]. There is genomic continuity between Later Stone Age people of southern Africa and the ethnographically documented KhoeSan [7–9]; archaeological evidence indicates both similarities and differences [7, 10–13]. Evidence of territoriality among Holocene foragers from the western and southern coasts of the Western Cape Province, shown through differential access to marine foods [14–17] is noteworthy, in part because of the apparent incongruity with ethnographic records. The dietary differences imply territoriality through perimeter defense, that is, exclusion of outsiders [5]. Diverse lines of evidence indicate that both social boundary defense and perimeter defense may be discerned in the archaeological record. One manifestation of the latter—community burial areas—has not previously been identified in the south-western Cape.

Genomic models suggest that the KhoeSan population was expanding throughout the Holocene [18]. It has been hypothesized that as population densities reach intermediate levels, cohabiting individuals’ interests are more likely to align and lead to actions excluding out-group competitors [19]. The establishment of a community burial place could be an example of such within-group cooperation. It is within this theoretical framework that we present evidence of funerary and burial practices by ancestral KhoeSan people who lived along the eastern side of Table Bay (around which the municipality of Cape Town is now located).

We have extensive evidence, gathered over many decades, of Late Holocene burial practices along the southern and western Cape coast [20–22]. Along the west coast, from Cape Town northwards, deceased individuals were buried soon after death, with the body placed on its side with legs flexed, knees close to the chin. Single interments are the norm. Personal belongings and other grave goods are rare. Although perishable items may have deteriorated, the absence of personal items suggests a different practice from that of Northern KhoeSan groups observed in historic times. There, observers recorded that when buried the deceased was clothed and adorned with beadwork as in life, wrapped in a kaross [22:121, 23]. Historic interviews with /Xam informants suggest that the spirits of the dead were believed to remain in the landscape, lending their support to the living [7]. Along the West coast, unlike other parts of the southern African coastline, single burials of newborn infants are rarely found. This suggests that deceased newborns may have been handled differently [24]. The presence of unadorned single burials across a broad landscape suggests that funerals were not elaborate rituals. Bioarchaeological studies of the individual skeletons have provided evidence of past foragers’ lives, and in a few cases, causes of death.

Death can result from actions that leave no mark on the skeleton. Most human skeletons from archaeological contexts provide no clues regarding cause of death. Nevertheless, forensic anthropology provides tools that allow discernment of damage to fresh bone, when the presence of resilient collagen and surrounding soft tissue modifies the bone’s reaction to external force [25]. Evidence of defensive wounds, puncture wounds and blunt force trauma has been reported from globally extensive times and places. Among the skeletons of South African Holocene foragers, there are several examples of accidents and misadventures from which people recovered [26, 27]. To date, healed cranial trauma has not been systematically studied, although cases have been reported [28, 29].

**Fig 1:**
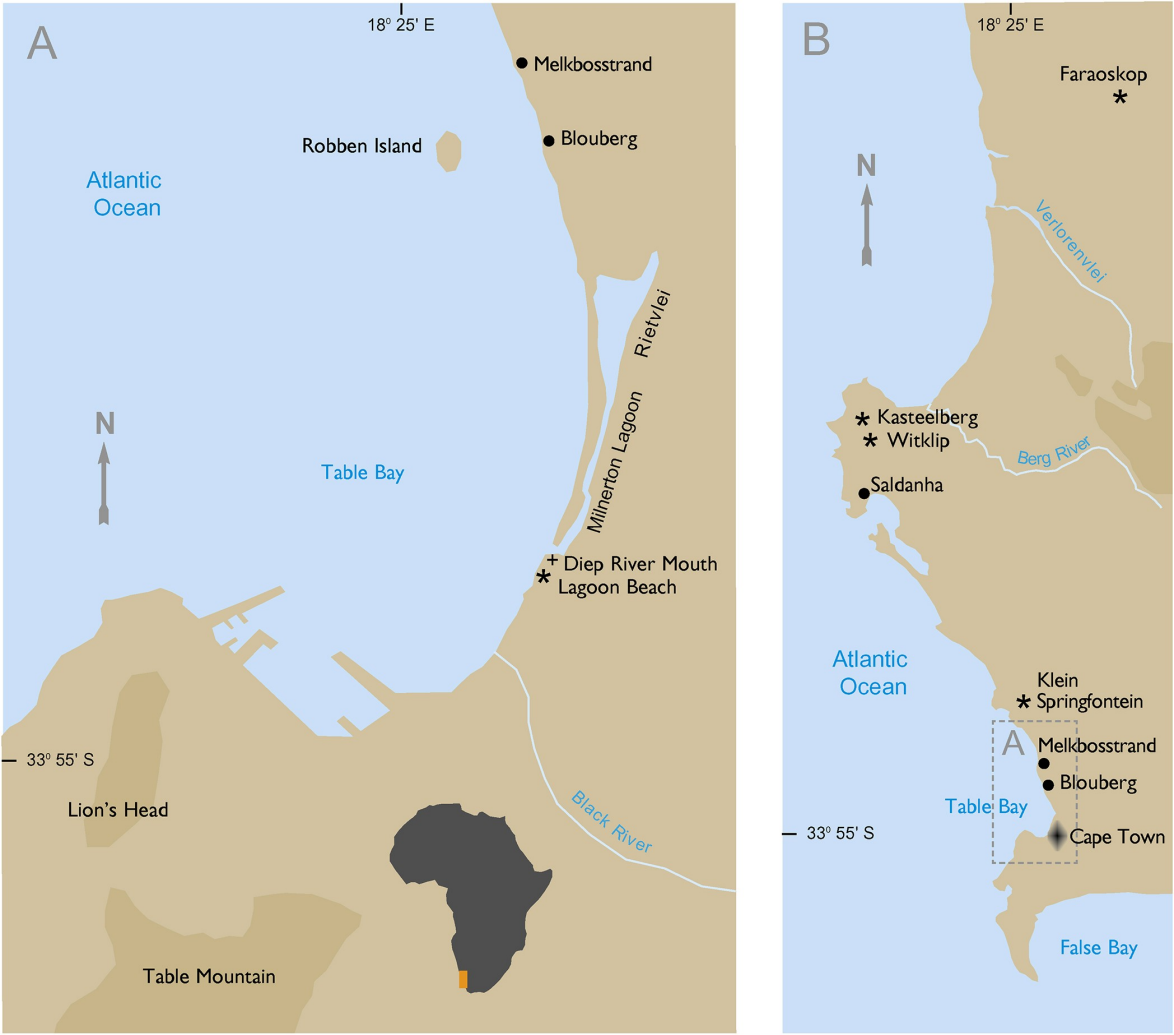
Map of the region, with key archaeological sites noted.

A number of instances of perimortem skeletal trauma have been documented from the south-western Cape [27, 30]. One site from the south-western Cape may represent an expedient burial of a group of murdered Late Holocene forager people. Of the twelve skeletons found at the Faraoskop Rock Shelter [32.07.31S; 18.36.52E) (Fig 1), five were excavated professionally [31]. Those five were flexed with no grave inclusions and all were missing their crania. The excavator concluded that the crania had been removed in antiquity. It has subsequently been suggested that their number, placement and bone breakage patterns may indicate that they were victims of a violent attack, later buried by surviving group members [32]. Dates of the Faraoskop skeletons cluster tightly around 2000 to 2100 BP [31, 33].

At another site in the region, Diaz Street, municipality of Saldanha, six individuals were buried separately, but very close together. One was found with a bone point within her chest cavity [34]. At Modder River, three children aged approximately 12, 6 and one year of age, all of whom had suffered major peri-mortem cranial trauma, were buried together [27, 35]. A double burial of an adult woman and an adolescent girl, both with cranial trauma, was recovered from Melkbosstrand [36]. There are a number of single burials with peri-mortem cranial trauma, summarized by Pfeiffer [27]. Nearly all date to 2500–2000 BP. A distinctive feature of these cases is the predominance of women and children.

The group of skeletons described in this report (including infants) form a unit by virtue of their tight geographical clustering, very similar radiocarbon dates and the presence of body adornment. We explore whether they have biological and cultural similarities. If they do, might they represent a cemetery used by a community over a number of generations? Given the prior reports from the broader region, is there evidence here for violent conflict?

### The Lagoon Beach site and a history of the discoveries

The littoral dune cordon running parallel to the eastern shoreline of Table Bay, though quite low in places, has had a major effect on river drainage into the Bay. From the north, the Diep (deep) River is diverted from Rietvlei southwards to run parallel with the coast for six kilometres, via Milnerton Lagoon, before entering the Atlantic. Similarly, the Black River, from the south, may at times have flowed about two kilometres northwards behind the dune cordon to join the Diep River and share its mouth (Fig 1). (The Black River has now been canalized and the mouth is further south.) The Black River drains the eastern side of Table Mountain, and together the two river systems provide the largest flow of fresh water into Table Bay. Although neither can be considered sizeable as rivers—the mouth today is dry during the summer drought—by local standards this estuary must have ranked as a major hydrological landscape feature. In the event of territorial rivalry, as hinted by the burial evidence, a feature like this river-mouth could well have served as a boundary between two territories. Reliance on the dense and predictable marine dietary resources of the coast [37] could be linked to competition for coastal access.

The site itself is approximately 200 metres south of the river mouth, at 33° 53’34” S, 18° 28’ 55” E. It is situated immediately inland of the beach, in dunes partly stabilized by vegetation. It extends over an area of about 10 x 10 metres. At this point we need to clarify the past and present use of place names. Much of the earlier documentation, discussed below, refers to ‘Milnerton Beach’. However, the Cape Town suburb of Milnerton has its southern boundary at the river mouth; the site itself falls within the suburb of Lagoon Beach.

Along this stretch of the Table Bay shoreline, a number of Later Stone Age burials were found in several discrete events from the 1970s to the 1990s. Bones were initially noted by non-archaeologists as they eroded out of the sand dunes. These were reported to archaeologists at the South African Museum (now Iziko South African Museums), and the skeletal and cultural material recovered was accessioned there. Files associated with Archaeological Data Recording Centre (ADRC) topographical map 3318CD provide some information supplementary to the Museum files. Some skeletons had ostrich eggshell (OES) beads, a feature that was immediately recognized as unusual for this region. Museum documentation indicates that in at least some cases, there were originally more beads than are now curated.

From 1996 onwards, there was extensive construction of housing immediately adjacent to the beach. This work triggered an archaeological survey of the area, which identified vestiges of shell middens (Phase 2 archaeological report, June 28, 1996, H.J. Deacon and R.J. Goosen). Earth moving associated with construction has re-shaped the surface topography of the area, both in the footprint of the buildings and in their surrounds. As a result, we can identify the approximate location of the burials but the archaeological context has been lost and the site destroyed.

There are other curated human skeletons labeled as coming from “Milnerton” and “Milnerton Beach.” These are not related to the group studied here. Those that have been dated are more recent, many within the last few hundred years, post-dating European colonization of the Cape (see Supporting Information 1). For example, UCT 321 certainly dates from the colonial era, as demonstrated by the extended burial position and associated buttons and pocket knife [38]. Locations (known for most individuals) are north of the area we focus on here, in Milnerton itself. In this study, we include all skeletons known to have come from Lagoon Beach, south of the Diep River mouth.

#### A chronology of burial discoveries at Lagoon Beach

The curated and documented materials are summarized in Tables 1 and 2. The earliest file (ADRC 05767, now in UCT Libraries as Iziko-05767, consisting of several records, as outlined below) describes events of October 1977, when a local youth (Adriaan Coetzer of Ysterplaat) reported to the Milnerton police a site of skeletal remains of two adult individuals. The remains were eroding out of clean sand of the littoral dunes, about 200 m south of the mouth of the lagoon. The police removed most of four individuals now accessioned as SAM-AP 6041A, B, C, D. The A, B, C, D notation reflects the association of the four individuals (with some co-mingling of skeletal elements as described below). Museum staff (Graham Avery) investigated subsequently and noted a “possible third individual indicated by part of a mandible found on the surface.” GA observed some thoracic vertebrae and one scapula *in situ*. From those torso elements, body positions of the adults were deduced to have been flexed on their sides. Impressions in the sand indicated that the adult skulls had been 70 cm apart. The adults were accessioned as SAM-AP 6041A and B, the letters being assigned arbitrarily. Skeletal elements of a child and an infant were recovered in proximity to the two adults; one was found beneath the adults, the other above (GA, pers. com.). They were accessioned as SAM-AP 6041C (remains including a partial mandible) and 6041D. Ochre staining of the sand and the presence of OES beads were noted. There is no notation regarding any grave shaft(s). The adult skeletons from this unit have been included in several biological anthropology research studies [39–41], but this report is the first contextual analysis.

**Table 1:** Lagoon Beach burials included in this study. All were discovered approximately 200 meters south of the Diep River (Milnerton) lagoon, eroding from coastal dunes. Information on adornment is from museum notes and extant holdings. Unless otherwise indicated, beads are OES. Within the 1997 unit, the catalogue unit that includes the skull is underlined. O = ochre; G.A. = Graham Avery.

Museum Accession Number (SAM-AP)	Date of discovery, Investigator, ADRC file	Age & sex	Radiocarbon Lab no.	Radiocarbon Date BP	δ^13^C ‰	δ^15^N ‰	%C	%N	C/N	Notes on human remains	Adornment Placement
**6041A**	1977, G.A. 05767 PART 1	VY Adult male	Pta-4768	2010±45^1^	-15.7	12.7	42.6	15.5	3.2		6041 group: OES on torso of one adult, +at unspecified locations
**6041B**	1977, G.A. 05767 PART 1	Adult male	Pta-4722	1800±50^2^	-12.0	15.8	39.7	15.3	3.0		
**6041C**	1977, G.A. 05767 PART 1	Child		Xx						fragmentary	
**6041D**	1977, G.A. 05767 PART 1	Infant		Xx						fragmentary	
**Not accessioned**	w/1970s group (G.A.)	Infant		Xx						‘Diep River baby’	None documented
**Not accessioned**	1979 05767 PART 2	L hip bone w/beads^3^ Child’s ulna^4^		Xx							Pelvic arrangement
**6083**	1983 SAPS	Adolescent, sex unknown	Pta-4358	2000±50^2^	-13.4	14.0	43.6	15.4	3.3	partial	Pelvic arrangement
**6085**	1983 SAPS	Child^4^	Pta-4224	1850±60^2^	-14.3	14.4	39.8	15.3	3.0	partial	Pelvis Arm/wrist L ankle Hand^6^
**Not accessioned**	1986	Infant		Xx						Fragmentary	Six perforated *Nassarius* shells
***1997 Unit*:**	G.A.										
**6420** **Upper Burial**	1992?	Adolescent, male?	OxA-36297	1860±31^5^	-12.9	15.0	43.3	15.6	3.2	‘Kit’ on chest; Headband likely; O on R hip bone; missing sacrum, sternum	R hand Pelvis & surrounds Waist Top of ilium Sand below pelvis Between R femur & tibia Neck & chest R humerus & femur
**6419/****21/****22 Indiv #1**	1992?	Adult female	OxA-36296	1841±29^5^	-15.4	12.6	43.4	15.6	3.2	Perimortem trauma to skull	Perforated shell Bone beads, torso L hand (7+) R hand (5+)
**6423/****6425** **Indiv #2**	1992?	Adult female	OxA-36298	1840±28^5^	-14.9	13.0	42.8	15.4	3.2	Missing scapula & humerus; OA at thumbs	R hand (wrist?) L hand (wrist?) Bone beads, torso OES from unspecified body regions
**6418****/6424 Lower Burial**	1992?	Adult male	OxA-36299	1892±30^5^	-13.8	14.0	43.5	15.8	3.2	Headband likely: O stain on R humerus; O nodule near L hand; missing sacrum, sternum	Several hundred beads: Beneath head (headband?) Necklace w/ochre R arm band w/ocher (Fig 2B) L arm band Upper chest String below pelvis Beads from on/within pelvis R hand
**Not accessioned**	1992?	Infant		xx						fragmentary	

^1^Date and stable isotope ratios reported here measured on femoral shaft. Cranium of this individual dated to 1824 ± 27 BP (OxA-V-2056-27) [42].

^2^ Date and stable isotope ratios measured on femoral shaft.

^3^Left hip bone of young adult, missing pubic symphysis, probable male (based on auricular surface), with multiple strings of OES beads across the ilium.

^4^Two units with partial remains of a juvenile, no overlap in elements, may represent the same child. The extant fragments are a small portion of what was originally excavated, as per museum notes and ADRC files.

^5^Date and stable isotope ratios measured on rib tissue.

^6^OES materials now missing.

**Table 2:** Selected cranial measurements and dental wear scores (as per 64) of the Lagoon Beach adult skeletons (mm).

	6041A	6041B	6083	6418 (lower)	6420 (upper)	6421 (Indiv1)	6425 (Indiv2)
**Max. cranial length**	179	X	172	174	X	X	179
**Max. cranial breadth**	133	140	137	133	X	140	x
**Zygomatic breadth**	119	x	X	124	X	115	x
**Basion-bregma**	132	X	X	127	X	126	126
**Basion-nasion**	96	x	x	103	X	94	96
**Nasion-gnathion**	105	107	X	110	X	107	96
**Mandibular body length**	101	105	X	98	90	104	98
**Mandibular body breadth**	107	x	X	116	80	94	95
**Symphyseal height**	34	32	X	28	30	34	29
**Wear score, M1**	2	6	2	7	2	7	6

An almost complete skeleton of a newborn infant was found slightly higher above sea level (more shallowly buried?), about 5 m from the two adults (G. Avery, pers com.). This is the infant referred to in Table 1 as the “Diep River baby.” It does not have an Iziko accession number, and so is referred to here as “unaccessioned.” There is no record or recollection (G.A.) of any beads or ochre with the baby. It is not clear exactly when, during the 1970s, the baby skeleton was excavated. Brief ADRC filings from November 1977 and 1978 appear to be related to subsequent explorations of the area, perhaps including the removal of this baby.

**Fig 2:**
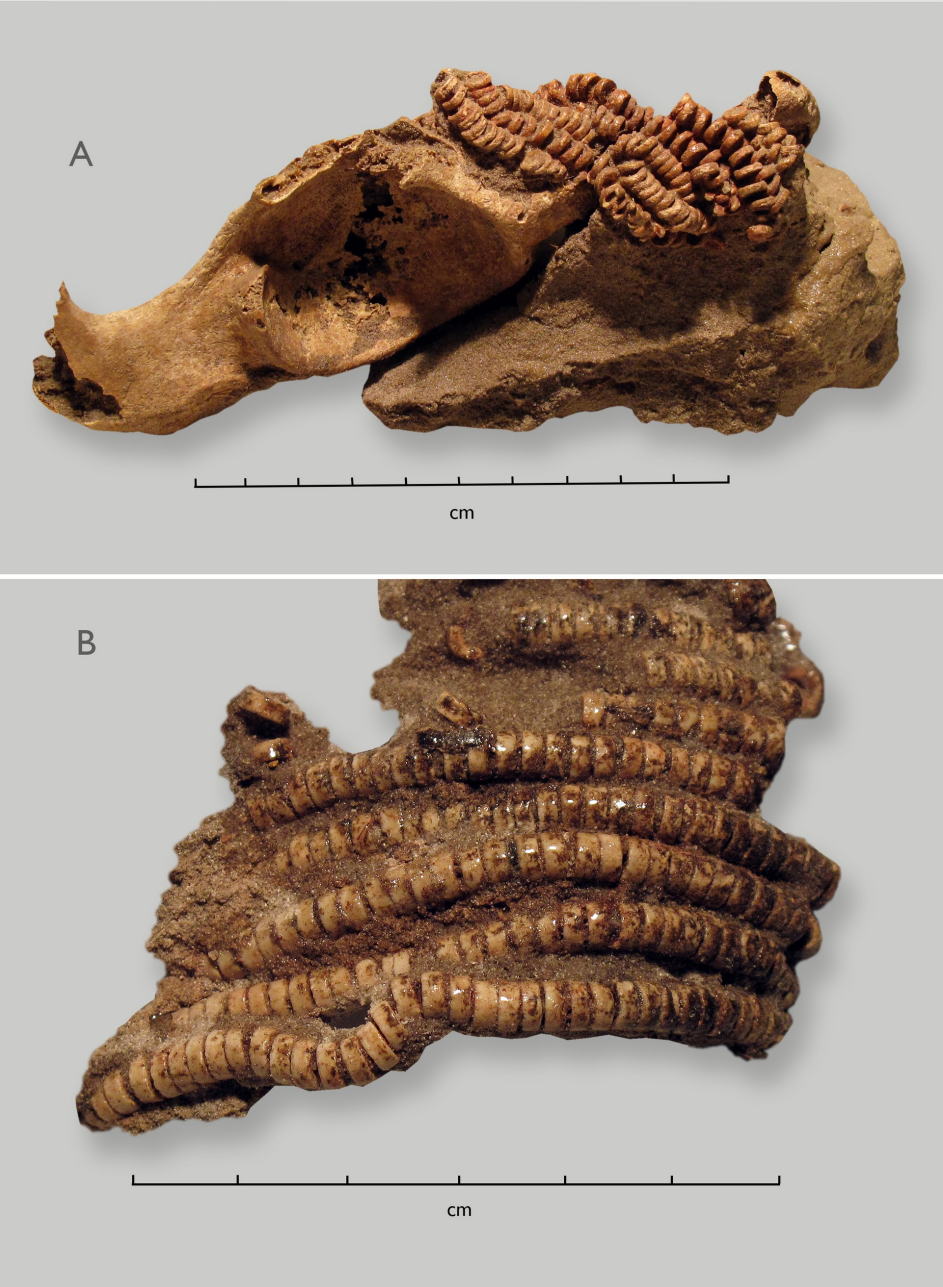
A. Left hip bone (unaccessioned) with multiple strands of OES beads, ochre-stained on bead surfaces. B. Armlet recovered with SAM-AP 6418—the lower burial of the 1997 unit—glued as discovered.

The 1978 site record form in the ADRC 05767 file says, “Additional skeleton. Skull and shoulder region removed by A. Coetzer. Many O.E.S. beads in hollow left [behind]. Some with ochre and still stuck together. Burial flexed on side. Fragment of preserved OES beadwork still on pelvis. Removed intact. (Still in ADRC for illustration).” We infer that this is the adult who is now represented solely by a partial left hip bone with beads (Fig 2). A juvenile ulna is stored in the same unit. These unaccessioned materials are described here for the first time. A sketch of the site of discovery was filed by G.A. and Hugh Walrond (16 Feb., 1978, ADRC 06055, now in UCT Libraries as Iziko-06055). It is annotated, “Beads under phalanges and metacarpals; beads at ankles and sacrum and about pubic area; stones and shell frags right through … [?]…area of disturbance where vertebral column and skull should have been. Many beads. Disturbance initially to edge of sacrum/pelvis. Skull frag on talus [slope] with beads. This possibly related to ..[?]… collected previously. Pelvis removed intact–bead work preserved. Immature individual; beadwork with some trace of ochre”. This site record includes a relatively detailed list of surface materials: “shell fragments, faunal remains, stone artifacts, OES beads and perforated pieces, Khoi potsherds and a sparse scatter of colonial era artifacts (fragments of clay pipe and Oriental porcelain).”

Two skeletons were accessioned in 1983, with the South African Police Services (SAPS) listed as donor, accessioned as SAM-AP 6083 and 6085. Museum records indicate only that the skeletons were found 200 m from the mouth of the lagoon. We are inferring that this means 200 m *south* of the mouth of the lagoon, thus the same locality as the materials described above. The site was not visited by Museum staff at the time of this discovery (G. Avery, pers. com.). A note in the box with the bones of SAM-AP 6085 refers to a complete skeleton, but such is not the case today. As with SAM-AP 6041, these skeletons have been studied in bioarchaeological research, but these are the first osteobiographic descriptions. Although accessioned at the same time, there is no documentation regarding their relationship *in situ*.

Other material from the 1980s include a small box of artifacts from Milnerton, dated 07.02.1986. Its contents are infant bones with six perforated *Nassarius* shells that may have been beads. This material is unaccessioned.

**Fig 3:**
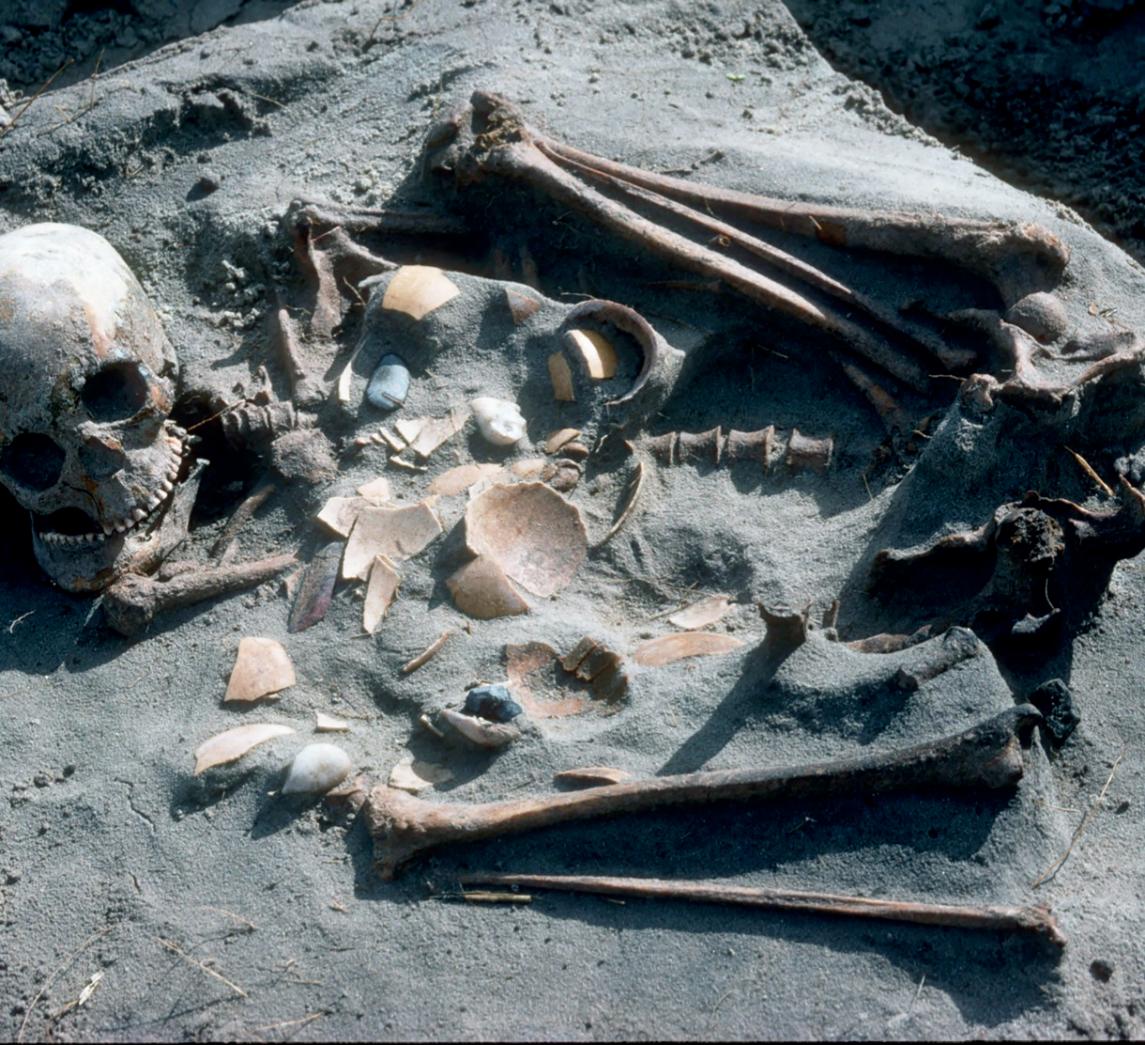
Field photograph (early 1990s) of exposed surface of “1997 unit”. Artifacts are clearly visible. Credit: G. Avery.

In the early 1990s, another burial was discovered by a member of the public and was once again followed up by Graham Avery. It, too, came from about 200 m south of the lagoon mouth, within at most a few meters of the prior discoveries. Because multiple skeletons were involved and it was relatively undisturbed, Avery chose to “jacket” the deposit in plaster of Paris and to transport it *en bloc* to the museum. There it remained unexplored until 1997, when two museum staff members (R. Yates and D. Stynder) excavated it. Regrettably, the photographic images and notes associated with the initial removal and the subsequent disentanglement have been lost. We have only one field photo (Fig 3) and a few notes that were placed in the boxes with the bones. R.Y. and D.S. labeled beads associated with specific body parts, and they sought to identify the distinct individuals. This report retains their labels. Four people were buried together. On the chest area of the top skeleton (Upper Burial), a late adolescent male, were artifacts that may have been held in a kit bag worn by the person or may have been tossed onto the bodies prior to covering them. Beneath this skeleton were two more-or-less parallel and somewhat co-mingled skeletons (Individual 1, Individual 2), both adult women. Beneath them was the skeleton of an adult man (Lower Burial). Various types and numbers of beads were associated with their heads, limbs and torsos. These individuals have been accessioned with numbers SAM-AP 6418 through 6425. They offer our best opportunity to learn about adornment and tool kits of the people buried in this small area on the shore of Table Bay.

## Materials and methods

The materials from the group burial excavated from the plaster of Paris jacketed block in 1997 (hereafter referred to as the 1997 Unit) were accessioned by Iziko in 2016. Permission for this study was granted by Iziko Museums on 10/18/16. Early in 2017, S.P. and L.H. applied standard osteobiographic methods to the human skeletal remains at Iziko identified as coming from “Milnerton.” With the aid of co-authors and museum staff, those skeletons found in the small area of Lagoon Beach were identified from within the larger collection. Particular care was taken to associate artifacts and adornments with specific bones, as far as possible, to note any evidence of ochre, and of perimortem damage to the bones. Age-at-death estimates were based on standard bioarchaeological methods [43–49]. Sex assignment favored pelvic criteria [50] supplemented by cranial characteristics [51] and other dimorphic traits [for example, 52]. Measurements and observations followed standard practice [51].

The 1997 unit had been boxed using from one to three boxes for each body, with some indications of uncertainty of association for some bones of the two women. Inventories for the four skeletons were completed, using indicators of age, sex and bone tissue quality. Particular scrutiny was directed toward distinguishing any bone breaks, scrapes or cuts that showed characteristics of the type of damage seen on fresh bone [25]. The crania and long bones of the 1997 unit and long bones of the other adults were CT scanned at Groote Schuur Hospital (exposure 120 kV, 250mAs; slice thickness 0.9mm), for biomechanical reconstruction [53] and to assist with exploration of any cranial trauma. 3D models were rendered (VG Studio Max 2.2, Volume Graphics) and assessed along with photographs. From CT images, bone volumes and distributions of humerus and femur shafts were assessed for their cross-sectional geometric properties (CSG) using the BoneJ plugin [54] for ImageJ (Rasband 1997–2012) to estimate torsional rigidity (*J*), maximum (I_max_) and minimum (I_min_) bending rigidity. The ratio of maximum to minimum bending rigidity (I_max_/I_min_) reflects diaphyseal shape (roundness) by comparing two non-fixed axes, indicating a bone’s capacity to resist bending forces [55]. Because different activities incur various degrees of long bone shaft bending, values generated for these variables reflect the nature and relative magnitude of habitual behaviours undertaken in life. Body size standardization applied the Ruff [56] method, so that values from the Lagoon Beach adults could be compared to those from other South African Holocene foragers [57]. That earlier study demonstrated a gender difference in upper arm asymmetry, with women more symmetrical than men, perhaps because gathering requires more balanced arm strength, and hunting methods rely on a dominant arm. Men from the forest ecosystem of the southern Cape showed significantly greater upper arm strength than men from the fynbos ecosystem of the south-western Cape, perhaps reflecting different hunting technologies. These are aspects of CSG we sought to explore.

Small rib bone samples were used for stable isotope analysis, cortical bone histology and radiocarbon dating (SAHRA permit 2519). Stable carbon and nitrogen isotope ratios were measured on bone collagen at the University of Cape Town (J.S.). Collagen was extracted using the whole bone demineralization method described by Sealy and colleagues [58]. Some stable isotope values are taken from previous studies [39, 59]; analytical details are provided in those papers. For samples measured specifically for this study, isotope ratios were measured on a Delta V Plus isotope ratio mass spectrometer, coupled to a Flash 2000 organic elemental analyzer via a Conflo IV interface (Thermo Scientific, Germany). Results are reported relative to Vienna PDB for carbon, and atmospheric nitrogen (AIR) for nitrogen. The standard deviation of repeated measurements of homogeneous materials was <0.2‰ for both δ^13^C and δ^15^N. Histological samples of rib tissue were assessed for age-at-death following the formulae of Cho and colleagues [60], applying the “ethnicity unknown” equation.

The artifacts associated with all the skeletons from the site were examined by J.S. and T.M. Maximum external diameters of the OES beads were measured by J.S., S.P. and Liesl Ward, using digital calipers. Diameters were recorded to the nearest 0.01mm.

Nine radiocarbon dates are available from measurements of human bone collagen of eight skeletons (Table 1). Given the difficulty of accurately determining the precise amounts of marine protein in coastal diets [61, 62], they were evaluated for possible contemporaneity through multiple approaches, using OxCal modeling and calibration software [v. 4.3, 63].

## Results

### The skeletons

Individual descriptions, including determination of age at death, sex and personal distinguishing features are provided here (see also Table 1). Skeletons are described in an order reflecting the chronology of their field discovery.

SAM-AP 6041A: The bones are in excellent condition. The hands and feet of this skeleton and that of 6041B are co-mingled. Identifiable as 6041A are the vertebral column, sacrum and hips, all major long bones, skull and mandible of a very young adult male. Sex is based on pelvic morphology but is reinforced by relatively prominent brow ridges and a nuchal crest. Epiphyses are partially open at late-maturing sites like the medial clavicle and iliac crest. The teeth are not very worn [stage 2 in the scheme of 64]. An age-at-death in the early 20s is consistent with all evidence. The occipital bone is divided (Inca bone). We noted no evidence of perimortem trauma, nor any pathological features.

Notes boxed with the unit indicate that “grave goods” were removed to be housed with artifacts. The ADRC notes (05767) mention flaked quartz and quartzite cobbles and a hematite nodule. We were unable to locate these. The ADRC notes (05767) state that “a few OES beads, some still linked and with ochre staining were found in situ very close to [three thoracic vertebrae].”

SAM-AP 6041B: Showing more post-mortem damage, especially to the skull, this skeleton is also quite complete. The complete pelvis and the prominent gonial eversion of the mandible indicate male sex. Morphology of the auricular surface and pubic symphysis suggest an age-at-death in the range of 35 to 45 years. Dental wear is greater than that of 6041A [64: stage 6], consistent with an older age. There is slight osteoarthritic (OA) lipping at most joints, but more developed OA on the joints of the right elbow and left knee compared to that on their antimeres. The lower spine shows remodeling from soft tissue trauma, suggesting herniated discs between lumbar vertebrae L2 through L4, and bone spurs suggesting a slipped disk at L4-L5. It seems highly probable that this spondylolisthesis would have caused back pain [65]. The bone of the superior sacrum is uninvolved. We noted no evidence of perimortem trauma.

SAM-AP 6041C and 6041D: There are numerous fragments of immature bones co-mingled with the 6041 unit. The child has been designated as 6041C and the newborn infant as 6041D. It is possible that these small bones had been spread across the area by natural processes, and that some immature elements found with other units represent the same people. The most diagnostic juvenile element is a left mandibular ramus in which the M1 is erupted, shows some wear on the lingual cusps, and root tips are complete, suggesting an age of 9 to 10 years [47]. The M2 crown formation is complete (suggesting an age of 7.5 to 8.5 years) but it remains unerupted. This suggests a median age-at-death estimate of about 9 years. The infant bones include ilium and temporal bones that can be assessed by size and maturity [48]. They are consistent with a death at less than about 4 to 6 months post-partum (i.e., an infant). These units are too fragmentary to assess for the presence of perimortem trauma.

Diep River baby (unaccessioned): Because of its well-preserved condition, curatorial staff chose to mount the skeleton for potential display. Individual bones are thus unavailable for measurement, and observations relating to age at death are limited. Based on the maturation of two visible tooth crowns (observed by S.P. in 2000), an age of 0 to 6 months post-partum is estimated. There is no indication of any abnormalities, and no *cribra orbitalia* (abnormally porous bone associated with anemia).

Other unaccessioned human remains from Lagoon Beach: Appearing to have been discovered in 1979 or thereabouts, a single incomplete left hip bone (*os coxa*) is notable for the multiple strands of OES beads positioned horizontally across the anterior ilium. These have been stabilized (glued) (Fig 2A). The bone is in poor condition. A small uneroded anterior portion of the iliac crest is fully mature. The pubic region is missing, but the auricular surface of the ilium is visible. Its surface is consistent with a young adult age at death. The absence of a raised auricular surface is consistent with male sex. Within this unit, housed with artifacts, is an immature ulna, with unfused proximal and distal ends. Its length, about 195 mm, suggests an approximate age at death of 10 to 12 years [49]. An unaccessioned box, dated 1986 and held with “artifacts from Milnerton Beach,” includes a group of small perforated shells and a few fragmentary bones from a newborn infant.

SAM-AP 6083: This skeleton is represented by the damaged, incomplete skull, mandible and postcranial skeleton of an adolescent. Features that are available to assess for age-at-death include a developing third molar (crown complete) and the vertebral rings (lumbar rings are fused, thoracic rings are not). Overall dental and skeletal maturation suggests an age of around 16 years [48], consistent with an observation of slight tooth wear [64, stage 2]. The skeleton is too youthful for histological age estimation methods to yield accurate results. Biological sex cannot be confidently ascertained. The fragmentary pelvis shows a flat auricular surface profile and a rather short superior pubic ramus. There are no septal apertures on the humeri. These features are male indicators. On the other hand, the palate is shallow, there are no brow ridges, and the distal humeri code as female in 3 of the 4 criteria, all relating to trochlear and olecranon fossa shapes [52]. There is bilateral, moderate *cribra orbitalia*. There is no evidence of perimortem trauma. A note with the skeleton, written in the 1980s, mentions that there had been a pelvic bead arrangement. There is a bag of OES beads with the skeleton, labeled “removed by A. Coetzer”.

SAM-AP 6085: This skeleton is represented by a few cranial fragments and lower limb elements of a juvenile whose age-at-death is too young to reflect secondary sexual characteristics. There are no teeth or jaw bones present. While this child appears slightly older than the child represented by 6041C, some mixing among these units is possible. Assessment of epiphyseal status [48] suggests an age around 12 years. There is no evidence of perimortem trauma. The notes with this unit suggest that the skeleton was quite complete when accessioned, with many OES beads.

A note with the skeleton indicates that grave goods were removed, to be housed with artifacts. They included OES beads from the pelvis, arm/wrist, the left ankle and under the hand. In 2017, only a few OES beads were curated with the skeleton, in small bags.

#### The 1997 unit

The one field photo shows the top skeleton (“Upper Burial”) to be on his back with arms and legs spread laterally (Fig 3). We have no information on the positions of the three lower skeletons. All four were close to one another, so that jacketing was feasible. Bone preservation of the four skeletons is mostly very good. Some bones from the torsos of Upper Burial and from Individual 1 appear to have decomposed subsequent to interment. The absence of the right scapula and humerus of individual 2 (despite presence of the complete right forearm and hand) suggests taphonomic disturbance (e.g. burrowing animals) resulting in the shoulder bones being separated, and hence left behind at the time of removal from the field. The absence of the right clavicle from Individual 1 is puzzling. The extent to which it was possible to assign the bones to individuals provides some clues about the juxtaposition of the bodies. RY and DS were careful to identify the anatomical sectors of each skeleton. Hands, feet, and ribs were separated by individual and bagged separately. However, there was some co-mingling in the boxes of postcranial bones from Individual 1 and Individual 2 (the two women), resulting in one box of bones that was unattributed to either person. This suggests that the two were very close to each other, perhaps intertwined to some extent.

While it is regrettable that there is very little documentation describing the removal and subsequent management of the 1997 unit, the material nevertheless provides a valuable view of people’s lives. Four people–three adults and an adolescent who was probably old enough to be considered an adult by the community–were placed in a single pit dug into beach sand. The completeness of the skeletons indicates that this occurred soon after death. While we have no information on the body position of the three people beneath the Upper Burial, the splayed position of the top person was unusual, and has not been reported for other KhoeSan ancestral burials. It may reflect an absence of funerary ritual, or burial in haste. A cluster of useful objects was found on the chest of the top person, some of which may have originally been contained in a bag. These include a tortoise carapace bowl, an ostrich eggshell flask, a fragmentary bone point/linkshaft, a hammerstone and some other lithics (Fig 3). They may be associated with this person, or they may have been in a bag belonging to any one of the people interred (see below). Anomalously large marine shells (see below) indicate that organic material was also placed there. Decomposition of the organic material may explain the deterioration of the spongy bone of the sternum and sacrum from two of the skeletons.

**Fig 4:**
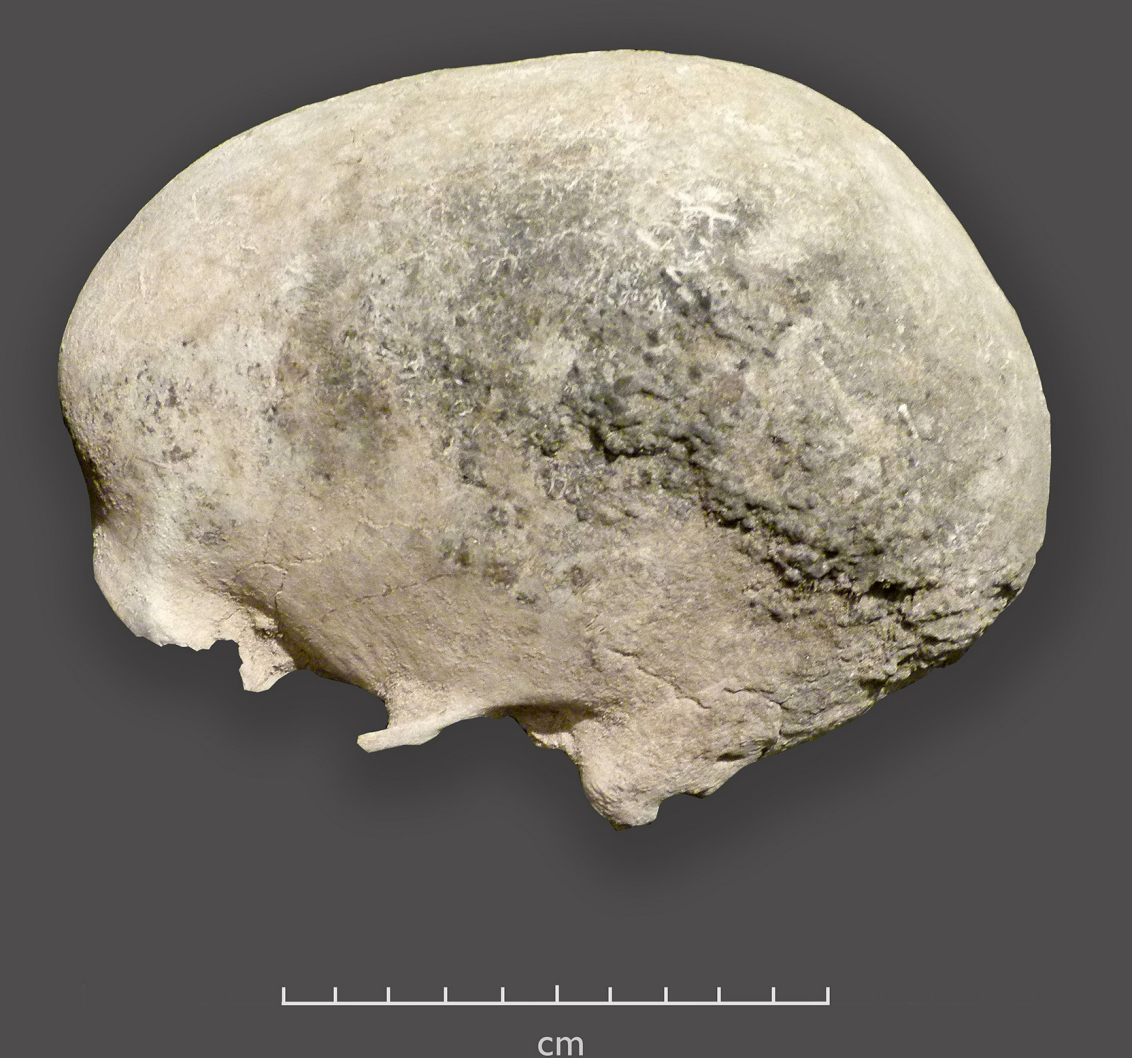
Left side of cranial vault, Upper Burial, SAM-AP 6420, showing possible hair in the area that would have been covered by a headband.

Upper Burial (SAM-AP 6420): This complete skeleton shows some postmortem damage to the long bones and their epiphyses, and some bleaching of some bones. The manubrium, sternum and sacrum are missing, and the anterior ends of the ribs have deteriorated. This is consistent with exposure to organic material on the chest. On the outside of the cranial vault there is a band of loosely adhering charcoal-colored material. It is woolly in texture and appears as little lumps. Its distribution and morphology are consistent with its being hair that was protected by an overlying headband. The preservation is better on the left side of the vault (Fig 4). The third molars were erupting, and their root development suggests an age at death between 16 and 18 years [47]. Patterns of epiphyseal fusion suggest an age of early to mid-adolescence [48]. The timing of dental and skeletal development, in which the former is ahead of the latter, is generally characteristic of males, in whom skeletal maturation occurs later than in females. The skeleton is too youthful for histological age estimation methods to yield accurate results. The skull shows male traits of high palate, large mastoid processes and a relatively prominent chin. While shape characteristics of an immature pelvis cannot be confidently relied upon as sex indicators, the morphology of this pelvis lacks female characteristics. Male sex is tentatively ascribed.

The many OES beads associated with this individual were separated into bags according to the body region where they were found: in association with the pelvis, neck and upper chest, right hand, and waist (also some more precise designations that probably relate to these same body regions, e.g. “Between right humerus and right femur, close to pelvis”, “sand below pelvis” and “Between right femur and right tibia close to pelvis”). Some beads from the pelvis are in strands, consolidated with glue. The absence of beads near the skull suggests that if there was a headband, it was not beaded. We did not see any evidence of pathological features or perimortem trauma to the bones.

Individual 1 (SAM-AP 6419/21/22): This skeleton is missing the sternum, sacrum, and right clavicle. Otherwise, the skeleton is complete. Bones from this skeleton were ascribed to three separate catalogue units, but paired elements were found among the boxes, supporting our assessment that they represent a single individual. There are no duplicated or extraneous elements. The pubic symphyses of this adult are at an advanced stage of remodeling, suggesting an age-at-death of more than 50 years. All the major cranial sutures have closed and the teeth are quite worn [64, stage 7]. The histological age estimate indicates a middle-adult age at death (40.5 ± 9 years). The skull shows a prominent chin and moderate brow ridge. Postcranial indicators are consistent with female sex. Both auricular surfaces (sacro-iliac joints) have unusual inferior faces, with concavity at the margins. This could represent accommodation of soft tissue cysts, although the bilateral occurrence is puzzling since these fluid-filled cavities are associated with localized circumstances [66].

Eleven OES beads have been glued by curators to two right metacarpals. Other adornments include at least six bird bone beads, plus bead fragments that may be associated with either Individual 1 or Individual 2 (body region unclear), and other OES beads from unspecified parts of the skeleton. Three perforated marine shells may have been used as beads or pendants.

**Fig 5:**
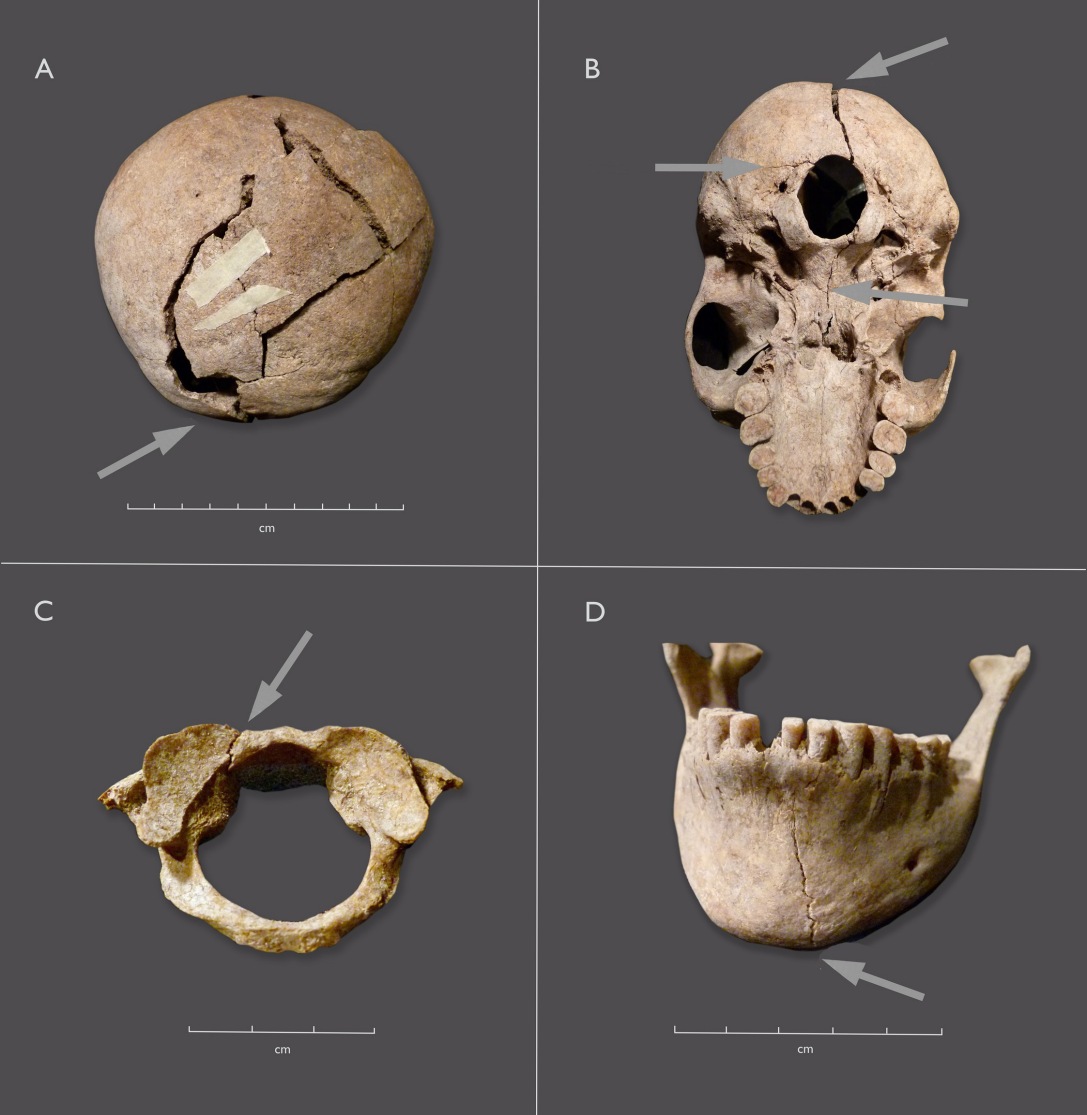
Individual 1, SAM-AP 6421, cranial trauma. A. Posterior view of skull, with locus of force indicated by white arrow. B. Inferior view, showing continuation of radiating crack through the cranial base. C. First cervical vertebra (atlas), crack indicated by arrow. D. Mandible, vertical crack through only the anterior body.

**Fig 6:**
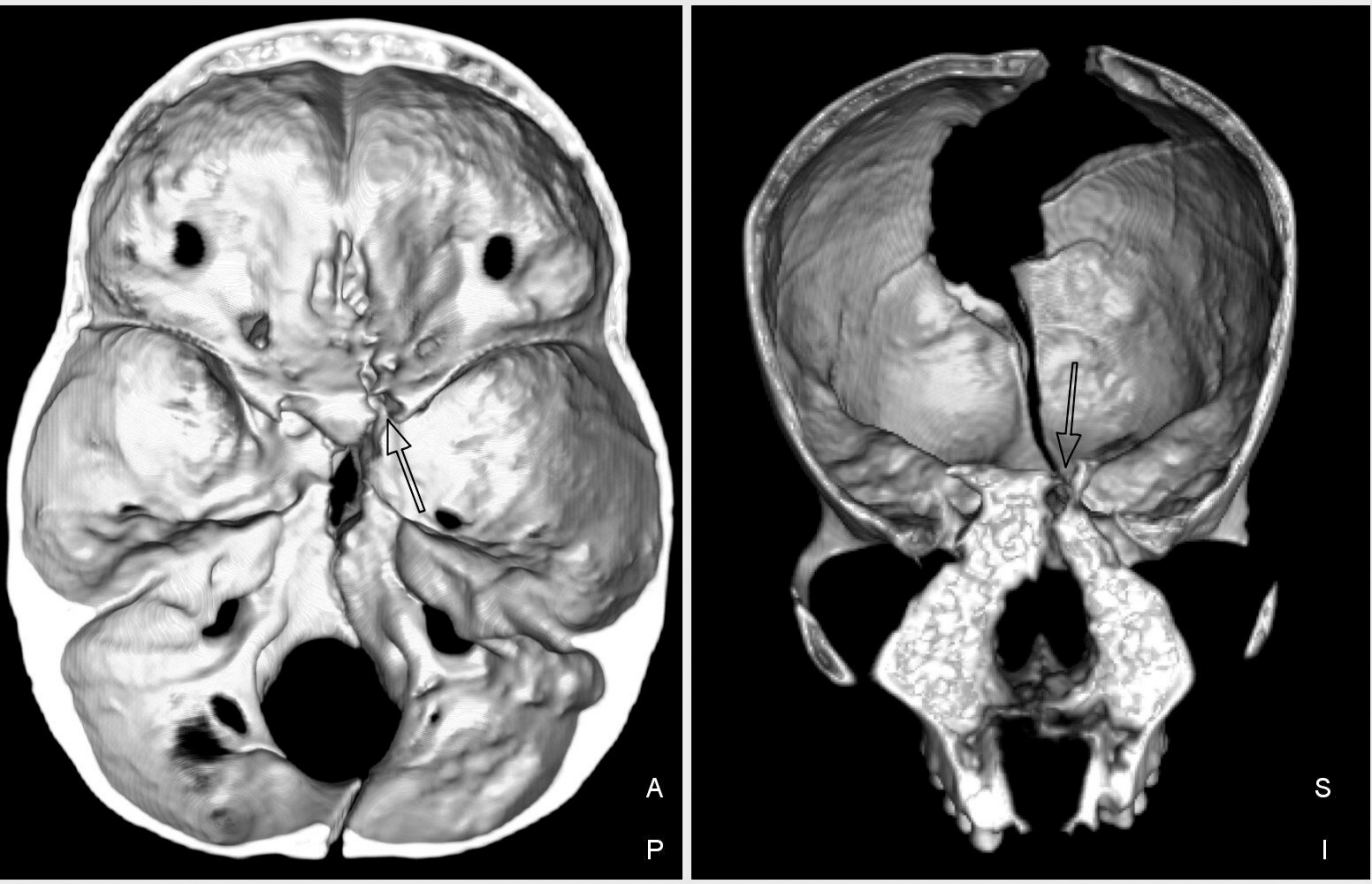
CT scan of Individual 1, SAM-AP 6421, showing cleaved sphenoid.

There is evidence of perimortem trauma in the form of fractures to the vault, cranial base, the front of the mandible and the atlas vertebra, all made when the bone was fresh (Figs 5 and 6). Some of the cracks to the vault have edges that suggest dry bone breaks, probably reflecting breakage from the weight of overlying sand. However, a pattern of cracks with beveled edges can be traced from a point of impact on the lower right half of the occipital bone (Fig 5A). The cracks radiate anteriorly (through the parietal, toward the frontal) and inferiorly (reaching the foramen magnum and proceeding through the basi-occiput (cranial base) (Fig 5B). Images from a CT scan of the skull (120 kV, 2550mAs, slice thickness 0.9mm) shows that the break continued, cleaving the *sella turcica* (body of the sphenoid) (Fig 6). A small fracture radiates laterally from the *foramen magnum* (Fig 5B). This is consistent with the distribution of force from the main fracture when it reached the *foramen magnum* [as per 67]. The cracks in the atlas vertebra and the front of the jaw (Fig 5C and 5D) could result from a blow abruptly forcing the skull forward and down, with the chin hitting the ground hard, or they could reflect a second blow. Anterior fractures of the atlas can be the result of hyperextension of the head and neck [25]. Such extensive damage could result from a fall from a significant height, with the back of the head landing on a protruding rock, the body then falling forward. The pattern and extent of damage could also be interpreted as evidence of a powerful blow that knocked the woman down, face forward. In the context of a mostly flat landscape with no rocky outcrops nearby and a group burial, interpersonal violence is the more parsimonious interpretation.

Individual 2 (SAM-AP 6423/25): This skeleton is of a particularly small adult, with cranial and pelvic morphology fully consistent with female sex. There are extensive ‘parturition scars,’ as have been observed among smaller females [68]. This may compromise the use of the pubic symphysis for age estimation. Age-at-death based on sternal rib ends and auricular surfaces is mid-40s, somewhat younger than Individual 1. This is corroborated by the younger histological age estimate for Individual 2, of 34.6 (± 9) years. Dental wear patterns are similar between the two women, but the degree of wear is less on Individual 2. The skeleton is in good condition, and is complete except for the right scapula and humerus, the left third metacarpal, and a few carpals and phalanges. Both of her thumbs show severe osteoarthritis proximally with pitting, osteophytes, and slight eburnation on one; both trapeziums are pitted, distorted in shape, with osteophytes. The fit between the metacarpals and the carpals is tight, suggesting compromised joint space and limitations to movement.

One bone bead is identified as having been near the vertebral column of Individual 2. There are nine OES beads from near the left hand and seven from the right hand, and other OES beads from unspecified parts of the skeleton.

While there is some breakage to the skull vault, the breaks appear consistent with dry bone. If the skeleton was lying on the left side, such breaks could occur from heavy pressure from above. We noted no evidence of perimortem trauma.

Lower Burial (SAM-AP 6418/24): This is an almost complete adult skeleton, except for some missing metacarpals and hand phalanges. Unlike the others in this group, several of the ribs are vertically split (post-mortem), which may reflect moisture damage. Pelvic indicators and those of the distal humeri are of male sex, although the skull and mandible have some equivocal features. Age-at-death, based on pubic symphysis and auricular surface, is in the late 30s to early 40s (mid-adult). Histological assessment of rib tissue is consistent with this, at 36.2 (± 9) years. The humeri are rather asymmetrical in size. Only the left has a septal aperture; the right has a prominent, sharp-edged ochre stain at midshaft, presumably associated with an object that was overlying it.

As with the Upper Burial, there is some dark material that appears to be hair and/or desiccated soft tissue adhering to the ectocranium (especially on the right side), consistent with areas protected by a headband.

The hundreds of OES beads found with this individual include concentrations from beneath the head, the neck, the chest, hip area (sacrum, pelvis), the upper and lower arms (both sides, see Fig 2B) and near the right hand. Only a few of the OES beads have visible ochre coloration. A piece of ochre was found next to the left hand. This may suggest that the left hand lay in proximity to the right upper arm, explaining the ochre stain on the right humerus.

There is one area of bone damage to the right parietal, about 30 mm from sagittal suture, 60 mm from lambdoid. The slight, oval-shaped depression is 12.5 x 22 mm and appears to have occurred when the bone was fresh. It may have been caused by a scrape against a rock. With this possible exception, we noted no evidence of perimortem trauma.

Other human bone fragments: Associated with the catalogued skeletons from the 1997 Unit were a small number of bones from at least one newborn infant. They include an ischium, components of two vertebrae and some digit shafts. They are at about the same maturational status as the infant bones of SAM-AP 6041D and the Diep River Baby, from an infant who died in the first six months of life.

### The artifacts

The presence of OES beads with the burials discovered in the 1970s and 80s is documented in ADRC records, Museum files, and in some cases by beads curated with the skeletons. We do not, however, have details of the relationships between the beads and the skeletons. The OES beads from SAM-AP 6083 are included in the assessment of bead size (below). There are also OES beads associated with the unaccessioned left hip bone (probably from a young adult man, shown in Fig 2A), and six perforated *Nassarius* shells found with a few bones of an infant in an unaccessioned unit, dated 1986. These shells would need to be carefully cleaned and the perforations examined under a microscope to find out whether they had been strung as beads. We did not do this.

The following items were discovered with the 1997 unit. In addition to the items listed below, there are other items preserved in the collection, including some large, unmodified marine shells. These may have been extraneous to the burial, but large shells were not found in the lower portions of the unit, so they may have been placed with the people interred. They include the white sand mussel *Donax serra*, found on sandy beaches such as Lagoon Beach, and various limpets (*Cymbula* and *Scutellastra* [formerly *Patella*] spp) and black mussels (*Choromytilus meridionalis*), which live on rocks. There are no rocky outcrops nearby, but similar shells can be found washed up on this stretch of beach today. The list below includes only items that are clearly identifiable as artifacts.

**Fig 7:**
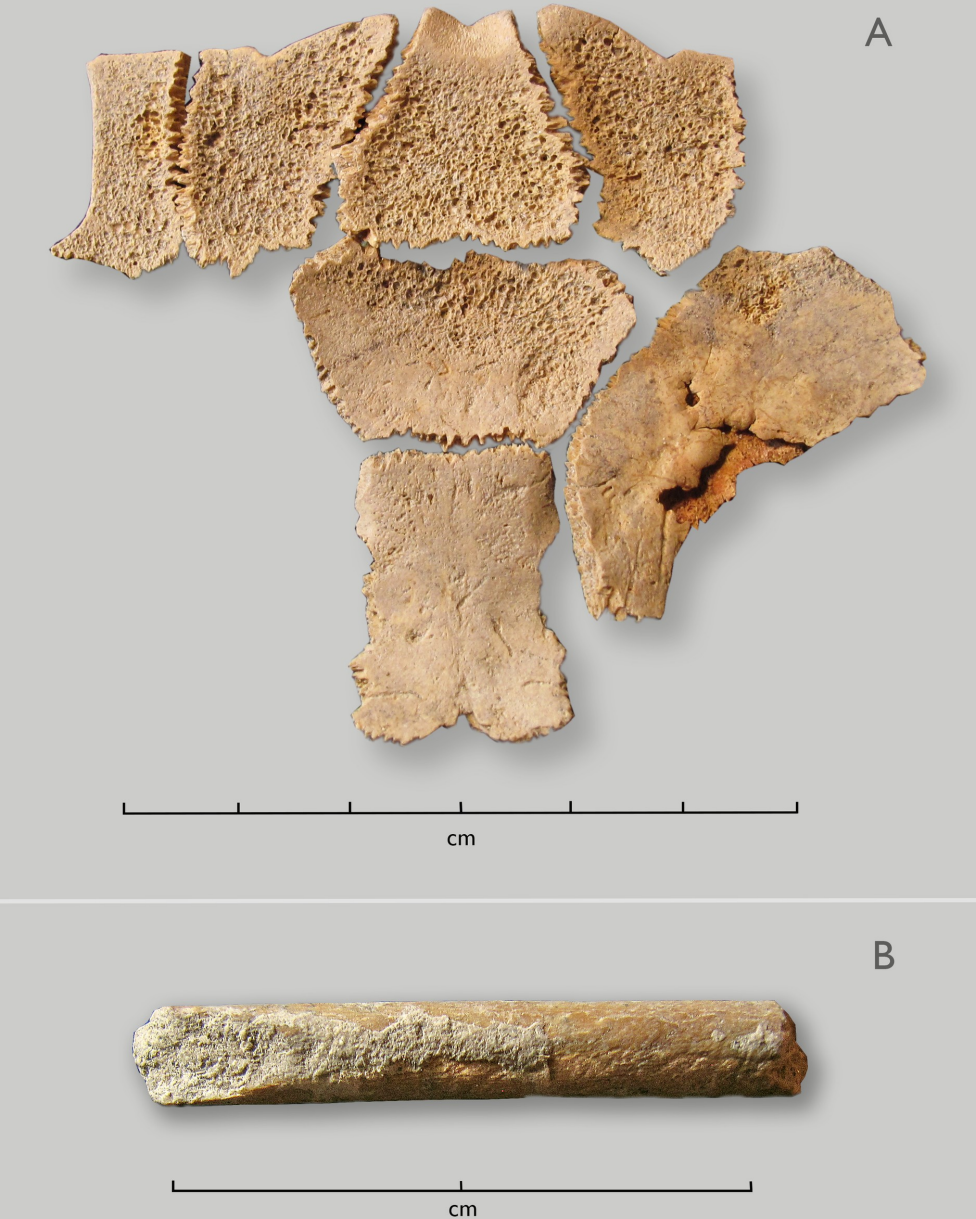
Artifacts from the “1997 unit”. A. Partial tortoise carapace bowl. B. Ground bone point or linkshaft, broken at both ends.

Items in the 1997 unit, all but the final one found on top:

1. Partial tortoise carapace bowl (7 plates): The set includes the nuchal plate and four adjacent plates, on which the margins are ground and smoothed, as is typical of tortoise carapace bowls. The inner surfaces have been ground down so that spongy bone is exposed. (Fig 7A)
2. Bone artifact fragment: This item is a fragment of a bone point or linkshaft, about 26 mm in length, 3.7 mm diameter with no discernable tapering, broken at both ends. There are striations on its surface, resulting from grinding and smoothing during manufacture. Given its fragmentary condition, we cannot tell if this was part of a projectile point, or if it might have been used to pin a kaross, or for some other domestic use. (Fig 7B)
3. A large part (possibly all) of a broken OES flask: There are more than 70 pieces, individually numbered, with fragment #5 preserving the aperture (which was 9.3 mm in diameter). There is no evidence of decoration.
4. Lithics:
  - A proximal fragment of large reddish silcrete flake (probably heat-treated) preserving patinated (possibly Middle Stone Age) flake surfaces. The piece was subsequently truncated and trimmed around the edges. The original bulb of percussion is preserved, but much of the original striking platform was removed by trimming. As noted in other Later Stone Age contexts [31, 69, 70, Fig 48a, 71, 72], this appears to be late Holocene re-use of a much older artifact.
  - A small cylindrical shale pebble hammerstone with hammering damage on both ends, and a flake scar on one end; dimensions are 48 mm in length, 26 mm by 17 mm in cross-section.
  - Quartz artefacts, including a small snapped quartz blade with edge damage on both lateral margins, quartz pebbles with a few flakes removed, flaked quartz crystal, a quartz cortical flake which could have come from one of the pebbles. If so, this may indicate flaking on site, although we do not have direct re-fits.
  - An ochre nodule, 25 by 15 by 7 mm, found near left hand of Lower Burial. It is red but does not streak.

#### Bone beads/tubes from the 1997 unit

Bird bone beads/tubes are much less common than OES beads. Two were associated with the right hand of Individual 2 (along with seven OES beads). The bones of Individual 1 and Individual 2 were co-mingled to some extent, especially the bones of the torsos. While some bone beads/tubes were identified from each of the skeletons, others may have come from either one. None show any decoration or ochre staining. Eleven could be measured. They vary in length from 9.1 to 16.6 mm (mean 12.9, s.d. 2.8) with maximum external diameters from 4.7 to 8.8 mm (mean 6.9, s.d. 1.6).

Ostrich eggshell beads: Most of the skeletons described here are associated with at least a few OES beads. Some individuals (especially the Lower Burial of the 1997 unit) were buried wearing hundreds. Most beads do not show ochre or other coloration. When present, ochre is visible mainly adjacent to the perforations, rather than on the external margins. This suggests that ochre was incorporated during manufacture and/or wear, not sprinkled onto the body at the time of interment.

Where the beads have been consolidated/glued to preserve their original configurations, they were threaded into strings (Fig 2A & 2B). We saw no evidence of beads assembled into panels, nor any “brick-work”-type patterns, as sometimes used when making headbands or aprons [73: Fig 4.21, 74: Fig 3.32].

**Fig 8:**
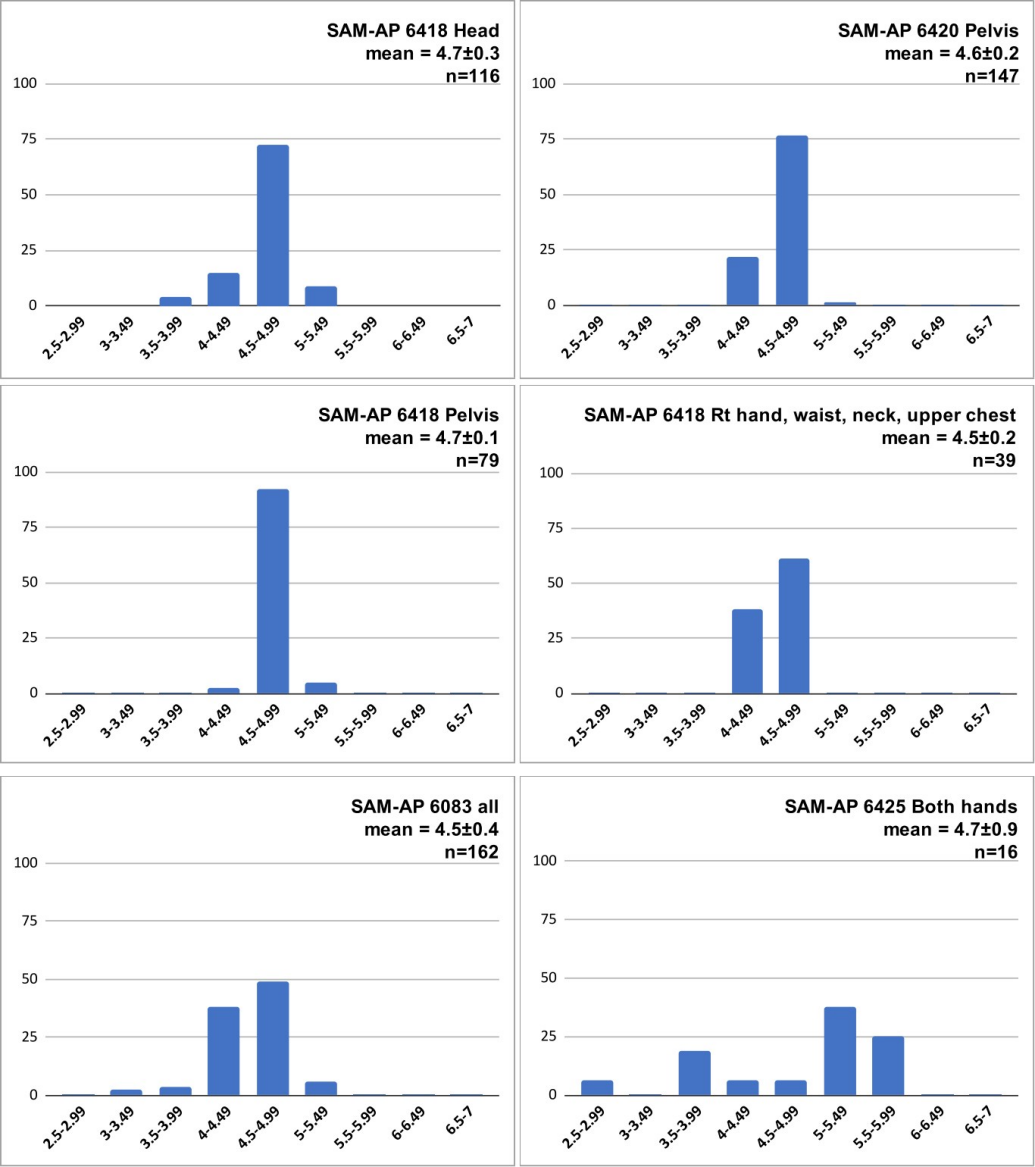
Histograms of maximum external diameters of OES beads: % of each sample per size category (mm).

We measured the maximum external diameters of OES beads associated with four individuals for whom the numbers of beads were sufficient to assess the average size and the consistency of size. Beads that were damaged or glued together were not measured. For two individuals, the Upper Burial (SAM-AP 6420) and the Lower Burial (SAM-AP 6418), we were able to measure beads associated with different parts of the body to find out whether different items of adornment were constructed from beads of different sizes. For SAM-AP 6420, we measured beads from the pelvis (n = 147) and the hand, waist, neck and upper chest area (n = 39). For SAM-AP 6418, we measured all 79 beads from the pelvic area and all 116 found under the head. We did not, however, measure the large numbers found in the neck area or around the arms. Based on visual inspection, these looked identical to the beads that we did measure. We also measured all the beads associated with SAM-AP 6083 that are still curated in the collection. Finally, we measured the 16 beads from the hands (wrists?) of Individual 2 (SAM-AP 6425), because these were visibly larger and more variable in size than those associated with the other individuals. Apart from these, the vast majority of beads measured had external diameters between 4 and 5 mm, both at different anatomical locations on single individuals, and across individuals (Fig 8).

#### Evaluation of the radiocarbon dates

The nine radiocarbon dates for the skeletal remains were produced in two laboratories: the Pretoria laboratory (laboratory identifier Pta-), measured using radiometric beta-counting techniques [75], and the Oxford Radiocarbon Accelerator Unit (ORAU; laboratory identifier OxA-), using an accelerator mass spectrometer [AMS: 76]. The OxA- AMS system yields more precise dates that the Pta- laboratory, with smaller errors. The acid-base-acid pre-treatment protocols of the labs were essentially the same and, given the relatively recent age of these samples, the measurements are equally reliable.

A complication for generating a precise age range is the lack of adequate estimates for the dietary proportion of marine foods among hunter-gatherers in this region, as radiocarbon from marine sources yields a substantial “reservoir” effect. Thus, the tissues of humans who relied on marine foods will appear “too old” at death. However, isotopic patterning of marine and terrestrial resources in this region complicates marine dietary estimations based on consumer δ^13^C and δ^15^N values [62]. While OxCal can accommodate broad uncertainties in the marine dietary proportion during calibration, this can result in modelled age ranges that are unhelpfully large. Assuming a marine dietary contribution of 15–45% for each individual, the dates have been calibrated using a *Mix_Curve* command to combine two curves, SHCal13 and MarineCal13, also accommodating the regional marine reservoir, ΔR [146 ± 85 ^14^C years: 77]. The calibrated dates, reflecting the large uncertainty in marine dietary composition, yield a 670-year range from 2000–1330 calBP, which is considerably broader than using SHCal13 alone.

**Fig 9:**
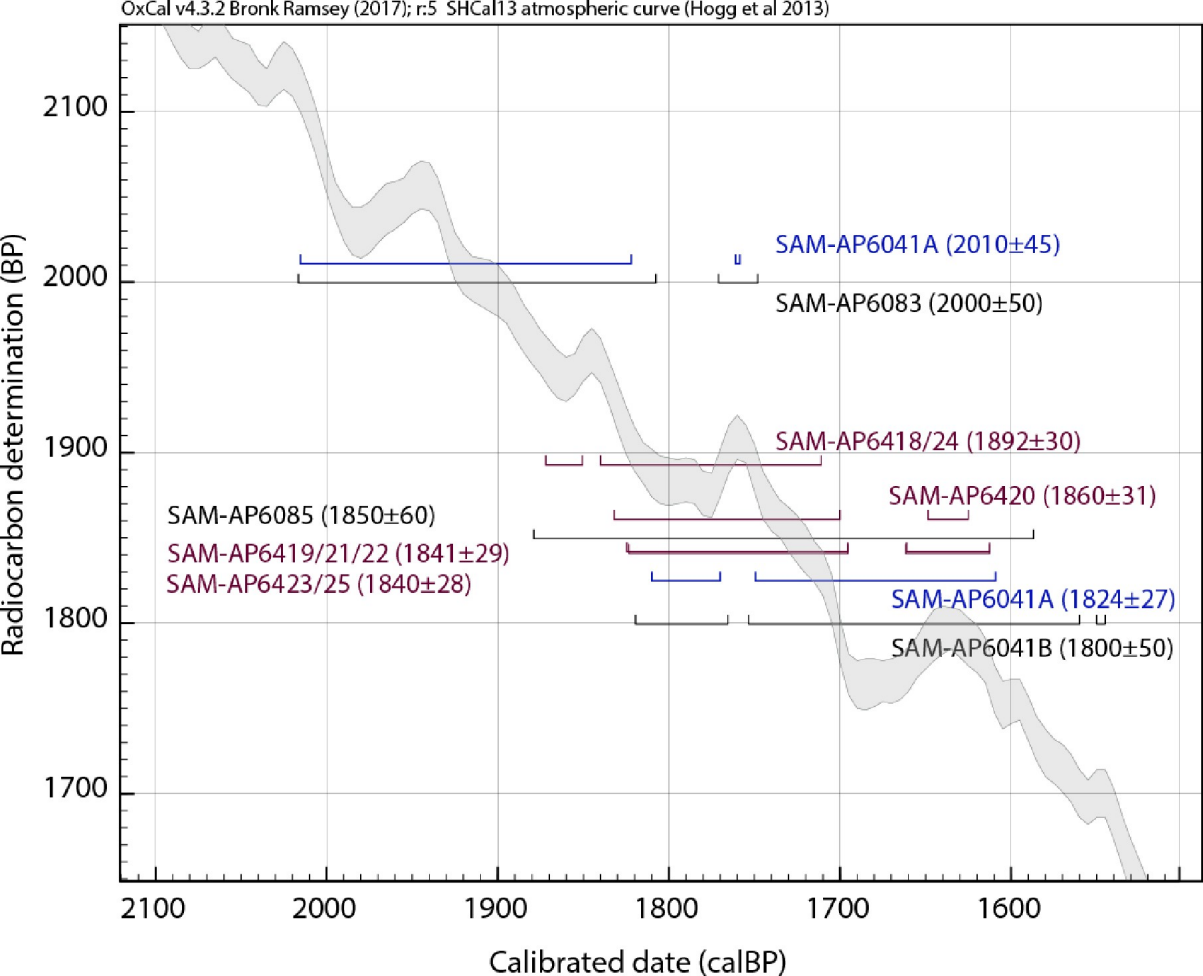
Nine radiocarbon ages for Lagoon Beach human remains. Calibrated using OxCal v. 4.3 and the atmospheric SHCal13 curve, shown in grey [78]. The two dates on individual SAM-AP6041A are in blue, and the four dates from the four individuals removed as a single unit in 1997 are in purple.

Two radiocarbon dates of one individual (SAM-AP6041A), one from each laboratory (Pta-4768, 2010 ± 45 BP and OxA-V-2056-27, 1824 ± 27 BP), illustrate how a broad date range may result, even within a single skeleton (indicated in blue in Fig 9). The full calibrated range for these two dates, using only the atmospheric SHCal13 curve, is 360 years (2020–1650 calBP). Although the calibrated ranges overlap, when the two dates are evaluated using OxCal’s *Combine* function, used to combine dates that derive from the same radiocarbon source, the function fails a Chi-squared test, indicating that the dates may not relate to the same event (df = 1, T = 11.10). We cannot explain the lack of correspondence between these dates.

The four dates of individuals excavated from the 1997 Unit can be successfully combined using the OxCal *Combine* function (df = 3, T = 1.73), confirming their close correspondence. Another method to evaluate contemporaneity amongst a set of dates applies the *Span* function, which estimates the length of a uniform phase. When the Unit 1997 dates are placed together in a *Phase* and the *Span* is estimated, the modelled difference spans from 0–160 years, or 0–170 when the dietary marine contribution is incorporated, indicating that it is within a 95% probability that the time between the first and last dates is zero, and so the dates may be contemporaneous (*A*_*overall*_ = >100, where *A*_*overall*_ > 60 indicates a reliable model) [79].

If all nine dates from the Lagoon Beach samples are analysed using the *Span* method, as above, the length is 0–302 years (*A*_*overall*_ = 69), although Pta-4768 (2010 ± 45 BP) is a possible outlier. If this date is removed from the analysis [following 80], the modelled *Span* estimate narrows to 0–208 years for all dates, widening slightly to 0–220 years if the marine radiocarbon contribution is included in the model.

#### Estimating the antiquity of the Lagoon Beach burials

**Fig 10:**
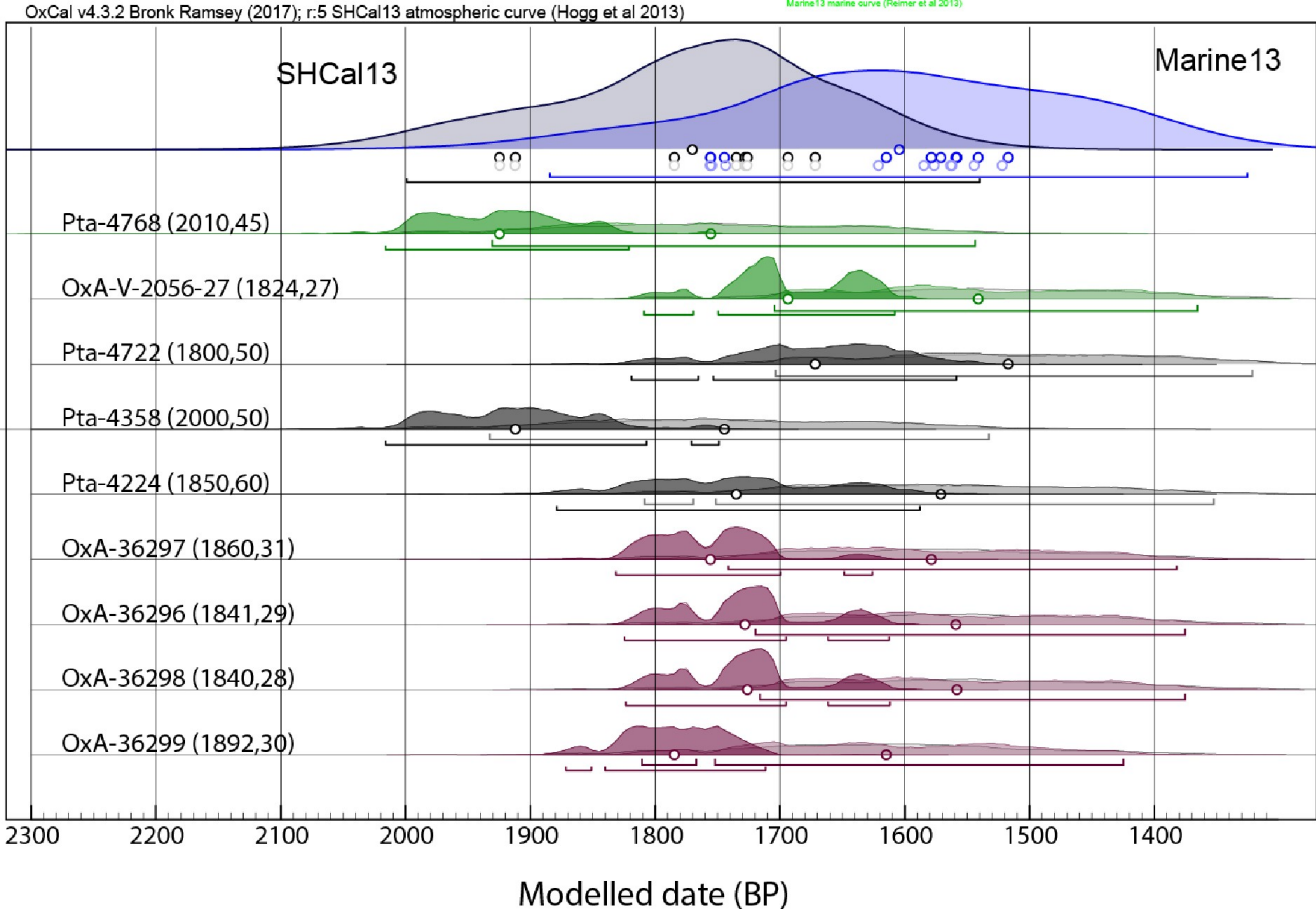
Nine radiocarbon ages for Lagoon Beach human remains. Calibrated using SHCal13 (dark curves) and a Mix_Curve with 15–45% Marine13 curve contribution with local ΔR of 146 ± 85 ^14^C years [77, 82]. The two dates on individual SAM-AP6041A are in green, and the four dates from the four individuals removed as a single unit in 1997 are in purple. The modelled KDE aggregations of all nine dates are indicated in blue.

The OxCal *KDE_Plot* function utilises Kernel Density Estimation (KDE) methods to aggregate dates and minimises the spread in age range that typically occurs when summing dates [81]. Applying this to the four dates of the 1997 Unit produces a possible age range between 1880–1620 calBP (μ ± 2σ), which shifts younger and broadens to 1790–1410 calBP (μ ± 2σ) when the marine dietary contribution uncertainty (15–45%) is included in the model. If all nine dates are aggregated using the *KDE_Plot* method, a modelled range of 2000–1540 calBP (μ ± 2σ) is suggested, which shifts to 1900–1340 calBP (μ ± 2σ) when the marine dietary contribution is incorporated (Fig 10).

These analyses confirm the impression of possible contemporaneity of the dated samples. They indicate a modelled age of between c. 1790–1410 calBP for the 1997 Unit, and a conservative range of between c. 1900–1340 calBP for all nine dates, given that a substantial marine contribution to the diet is likely. The full range for the uncalibrated dates from the Lagoon Beach samples, including errors, spans 305 years, and once calibrated (using the atmospheric SHCal13 curve), the full probability range for all dates is 470 years.

### Behavioral indicators of the Lagoon Beach group

#### Diet from isotopes and tooth wear

Values of δ^13^C_collagen_ and δ^15^N_collagen_ for adult skeletons are listed in Table 1. Values for δ^13^C range from -15.7 to -12.0‰, δ^15^N from 12.6 to 15.8‰. The values from the two men in unit SAM-AP 6041 bracket almost the entire range seen in the group. The group values fall within the range of values previously documented for precolonial coastal skeletons from this region (δ^13^C from -17.9 to -10.6‰, δ^15^N from 10.2 to 17.7‰) [39, 83]. This is a large range of variation with the more positive values indicating heavier reliance on seafood; the most positive probably reflect marine specialists. There is chronological patterning in the regional dataset, but we do not see any patterning within the eight individuals reported here. We can confidently say that marine foods made up a substantial proportion of their diets.

Previous studies of the teeth have shown that consumption of marine foods is positively correlated with heavy occlusal wear [33, 39] because marine foods tend to be gritty. People who died in childhood or young adulthood, like SAM-AP 6041A, 6083 and 6420, have modest levels of wear on their permanent molars, including some flattening of cusps on first molars [scored as 2, as per 64]. By middle adulthood (ca. 30–50 years)—represented by SAM-AP 6041B and Individuals 1 and 2 from the 1997 Unit—the M1 and M2 cusps have flattened, in a wear pattern consistent with the grinding motions of chewing (scores are provided in Table 3; see maxillary wear in Fig 5B). The adult skeletons vary with regard to the amount of calculus adhering to the teeth. Within the 1997 Unit, Individual 2 and the lower burial both have calculus deposits, while the other two do not. There are no instances of pre-mortem tooth loss, no caries and no abscesses. There are no hypoplastic defects visible to the naked eye. There is no evidence of wear patterns consistent with use of the teeth in non-masticatory activities, nor with dental hygiene. The dental evidence is consistent with the isotopic evidence that marine foods were important in their diets.

**Table 3:** Selected post-cranial measurements of the Lagoon Beach adult skeletons (mm). Values for SAM-AP 6083, an adolescent, are from long bones with fused epiphyses. Values for SAM-AP 6420, a younger adolescent, are estimates that include the yet-unfused epiphyses.

	6041A	6041B	6083	6420 (upper)	6419/22 (Indiv1)	6423 (Indiv2)	6424 (lower)
**Max humerus length**	R: 290	R:311	X	R:261	R:273	X	R: 292
	L:285	X	X	X	L: 271	L: 263	L: 285
**Humerus head diameter**	R: 37.8	R: 40.7	X	X	R: 34.7	X	R: 36.9
	L: 35.7	X	X	X	L: 34.4	L: 32.9	X
**Max ulna length**	R: 250	R: 262	X	R:212	R: 223	X	R: 241
	L: 250	L: 261	L: 233	L:217	L: 222	L: 214	L:236
**Max clavicle length**	R: 155	X	X	R:119	X	R: 116	R: 154
	L: 146	L: 140	L: 125	L:117	L: 138	L: 116	L: 153
**Max femur length**	R: 415	R: 432	R: 394	R:417	R: 403	R: 396	R: 421
	L: 413	L: 432	L: 391	L:411	L: 401	L: 394	L: 425
**Femur head diam.**	R: 40.6	R: 42.2	R: 37.6	X	R: 38.8	R: 35.6	R: 41.2
	L: 40.5	L: 42.1	X	L:37	L: 38.4	L: 35.6	X
**Max tibia length**	R: 365	R: 374	R: 336	R:313	R: 343	R: 331	R: 367
	L: 371	L: 380	L: 333	L:312	L: 343	L: 331	L: 367
**Bi-iliac breadth**	219	217				192	

#### Skeletal size and shape, and behavior from biomechanics

Selected craniometric values for the adults are presented in Table 2. They are consistent with those of the KhoeSan ancestral population [84–86]. The dimensions of the adult long bones are consistent with values from other coastal foragers [87] (Table 3). The tallest adult, SAM-AP 6041B, would have been about 1.6m [formula from 88]. The adolescent with fused femoral epiphyses, SAM-AP 6083, would have been the shortest, at about 1.5m. Among the five adults, the three men were taller than the women, and they have larger femoral head diameters which reflect greater body mass (male mean = 52.6 kg, female mean = 43.1 kg; [formula from 89]). Crural and brachial indices are consistent with expected values for the KhoeSan ancestral population [90].

The paired humeri of three adult males and one adult female, as well as one un-paired humerus from a second female, were analyzed for cross-sectional strength properties. (Table 4). As a group, the males have upper limb strength values that fall within the expected range for Later Stone Age males, falling closest to those from the fynbos biome [as per 57). All three males have higher torsional rigidity in the right distal humerus than the average for fynbos males. The Lower Burial from the 1997 unit is distinctive for his high degree of arm asymmetry which is more characteristic of males from the forest biome. The two adult females have arm strength measures in line with other Later Stone Age females, although both are below the mean for fynbos females, with Individual 1 being more gracile, like females of the forest biome. Only Individual 1 preserves both arms, from which we can see the characteristic low level of asymmetry that has been observed in Later Stone Age females relative to males [57). Femora from three males and two females were assessed for torsional strength at midshaft. The Lagoon Beach adults tend to have gracile lower limb robusticity compared to other Later Stone Age adults, with two of the three males and both females having midshaft femur torsional rigidity values below the mean. SAM-AP 6041B stands apart from the others with relatively high lower and upper limb robusticity (Table 5).

**Table 4:** Cross-sectional geometric properties of Lagoon Beach adults, compared to mean values of Later Stone Age adults of the fynbos and forest biomes (as per 57). Values of *J* for distal humerus shaft and mid-shaft femur are body-size-standardized as (body mass*bone length)^2^*10^5^ [56]; humerus asymmetry is absolute value of unstandardized *J*.

		Humerus 35%			Femur 50%
		*J* Right	*J* Left	% Asym	*J*
**Fynbos Males**		145 (n = 5)	111 (n = 4)	18 (n = 6)	388 (n = 10)
**Forest Males**		138 (n = 5)	124 (n = 6)	32 (n = 6)	383 (n = 11)
**All Males**		141 (n = 10)	119 (n = 10)	25 (n = 12)	391 (n = 21)
	**Lower Burial (6424)**	154	110	46	336
	**6041A**	149	163	5	339
	**6041B**	172	139	24	442
**Fynbos Females**		117 (n = 5)	106 (n = 6)	10 (n = 5)	292 (n = 9)
**Forest Females**		98 (n = 9)	92 (n = 10)	12 (n = 12)	334 (n = 15)
**All Females**		105 (n = 14)	98 (n = 16)	11 (n = 17)	318 (n = 24)
	**Individual 1 (6419/21/22)**	93	92	3	304
	**Individual 2 (6423)**	-	104	-	281

**Table 5:** Mann whitney U comparisons of Lagoon Beach males and females to Later Stone Age fynbos biome comparators [57] (p-values). Distal right humerus and midshaft femur body-size-standardized *J*.

	Milnerton Males (n = 3)		Milnerton Females (n = 2)	
	Humerus	Femur	Humerus	Femur
**LSA Males**	0.371	0.870		
**Fynbos Males**	0.786	0.937		
**LSA Females**			0.933	0.615
**Fynbos Females**			1.000	0.727

## Discussion

The human skeletal remains described here represent people who were buried on a small patch of Lagoon Beach, probably during a relatively brief period of time. Their radiocarbon dates are similar and most individuals (all for whom we have substantial descriptions) were buried with OES beads and sometimes other items. This is very unusual in the context of the south-western Cape region as a whole, which leads us to infer substantial cultural similarity among those who were buried here. The remains include infants, at least two of whom were less than one year old, who were not clearly interred with other persons. While we have reported the skeletons in units that reflect discrete discoveries, the presence of fragmentary infant and juvenile bones with several adults suggests that this section of beach may have held additional burials that were disturbed long ago. Hence, the demographic features of the extant units support a conclusion that this was a locale where people of various ages and both sexes were interred over a time period that may have represented some number of generations.

### The presence of infants

Despite the likelihood that in any human society some newborn infants will not survive [91, 92], relatively few skeletons of infants (less than one year of age [48]) are discovered archaeologically. In a survey of curated Later Stone Age immature skeletons, the ratio of infants to older juveniles is about the same from the western fynbos (7 of 23), the afromontane forest (17 of 60) and the region east of the forested area (8 of 51) [24]. In all regions, most of the infants were found in rock shelters. Most of those found in coastal dunes were interred with adults. The previously reported infants from the western fynbos include four from rock shelters: Steenbokfontein [93], Rooiels [94] and Byneskranskop ((2, one of them with pathological features [95] [96]). One was found in an open context, buried with a young woman who had been killed (Quoin Point [97]). There is scant information on age-at-death of this latter infant who may have died *in utero*, and whose remains are now lost. It may have been prenatal. Another duo comes from Mossel Bay, discovered in sand dunes in 1925 [98] (SAM-AP 4312 and SAM-AP 4313). Museum notes say the “young child” was found together with the adult. Dental formation suggests that infant died in the first six months post-partum. The skull of the adult was judged to be female [42] and was dated to 2260 ± 170 BP (Pta-2164) [98]. We have now added to this list of infants the “Diep River baby” and partial remains of at least one additional infant from Lagoon Beach, represented by the partial skeleton of SAM-AP 6041D and fragments of approximately the same maturity that were found in two other contexts: the 1997 unit, and as part of a 1986 discovery. The latter bones are associated with perforated *Nassarius* shells. There is no overlap of elements among these three find spots. It is possible that they all represent the same infant, although it is not obvious what taphonomic factor would spread the small, delicate bones so broadly. Based on current evidence, this locale is the only open-air context in the south-western Cape where independently interred newborns from the Later Stone Age have been found.

### The significance of the beads and beadwork

Ethnographic records of twentieth-century hunter-gatherers from southern Africa agree that OES beads were made by women [99:98, 100:9, 101]. They were worn by both men and women, although apparently more often, and in larger numbers, by women [100, 102, 103]. At Lagoon Beach, beaded items were associated especially with men. In the 1997 Unit, evidence from the presence of desiccated tissue on two of the crania and OES beads from the cranial region of one suggests that the two men were both buried wearing headbands. Both also had large numbers of OES beads, especially but not exclusively around their hips. There is a single male hip bone from this section of beach that had multiple strands of OES beads strung around the hips (see Fig 2A), and notes indicate that one of the men from the SAM-AP6041 group had OES beads on his torso. There are also notes that OES beads were associated with the pelvis of the adolescent SAM-AP 6083 (of undetermined sex). Perhaps these features, of headbands and pelvic adornment made of OES beads, represent characteristic men’s garb.

The women in the 1997 Unit had fewer beads, but both had bird bone beads associated with their bodies. The OES beads were associated with their hands/wrists. We note, however, that there was co-mingling of the women’s skeletons. There was no evidence for headbands or pelvic aprons associated with the women’s skeletons.

Some twentieth-century records report the use of different-sized beads in beaded items worn on different parts of the body [100]. At Lagoon Beach we see little evidence for this type of differentiation, although the only sample with a significant proportion of large beads (>5mm external diameter) was the small group (n = 16) associated with the hands of SAM-AP 6425.

Silberbauer [101:227] reported that an apron measuring 22 by 28cm and containing 4000 beads represented nearly 200 hours of work. Bleek [100] reported that Naron women prized beads and beaded items highly and as they grew old, handed them on to their daughters. The beads interred with the Lagoon Beach individuals were surely similarly valued. Consigning them to the graves–especially in such large numbers–speaks to cultural values and burial practices very different from those reflected in most late Holocene west coast burials, which usually lack grave goods of any kind. Rare exceptions include the report by Orton and colleagues [84] of decorative shell artifacts associated with a male skeleton dating to 2120 ± 60 BP (TO-8952; SAM-AP 6383) from Blouberg (*c*. 33° 47’ 58”S 18° 27’ 41”E). A double burial dating to 2970 ± 60 BP (Pta-8807) with OES beads, bone beads and shell beads was excavated at Kleine Springfontyn (33° 38’ 7” S, 18° 26’ 37” E) (SAM-AP 6317A and B; R. Yates, unpublished manuscript). The more complete skeleton is that of an adult male [42]. Both these find spots are relatively close to Lagoon Beach (Fig 1); it is interesting that Kleine Springfontyn dates to approximately a thousand years earlier.

### Who were victims of violence?

On at least two occasions, members of the Lagoon Beach group were buried together, having died at more-or-less the same time, at a nearby locale. In the 1997 Unit, the uncharacteristic splayed position of the upper skeleton suggests exceptional circumstances at his time of death and burial. We interpret the cranial trauma noted in Individual 1 as evidence of interpersonal violence. If this is the case, the injury provides no clue regarding where the conflict took place, but the placement of four complete bodies in one shared burial suggests that the deaths occurred close to where the skeletons were found. It is improbable, though perhaps not impossible, that a hazard like neurotoxic shellfish poisoning or a disease abruptly killed them all. If the deaths resulted from aggression, the context and the combatants remain unknown.

The SAM-AP 6041 group also consisted of at least four individuals: two adult men, a child and an infant. These skeletons were not recovered by archaeologists, and some evidence may have been missed. Here, we have no definitive markers of violence, but the group burial is suggestive. Globally, interpersonal violence among small-scale communities has been conceptually linked to phenomena like warfare and massacres [104, 105]. Although interpersonal violence has been a focus of many studies [106–108], few report evidence from immediate-return hunter-gatherers. Analyses of ethnographic data suggest that when lethal aggression occurs, the basis is most commonly homicide, less frequently feud, and rarely war [109, 110]. However, archaeological evidence of inter-personal violence among putative hunter-gatherers has been reported from more northerly African locales [111–115]. A meta-analysis of thousands of crania of hunter-gatherers who lived in what is now central California, 1,530 to 230 cal BP, provides a potentially useful parallel to the south-western Cape. Using archaeological data about resource availability and population density, competing explanatory hypotheses of sociopolitical complexity and resource scarcity were juxtaposed against instances of blunt-force and sharp-force perimortem damage. Perimortem sharp-force trauma to the skull was best predicted by resource scarcity. Neither sharp- nor blunt-force trauma were linked to the development of more complex hunter-gatherer adaptations [116].

Despite careful scrutiny, we noted no evidence of perimortem damage on the bones of any individual, other than one person in the 1997 Unit. There, Individual 1 provides evidence of sudden death from a forceful blow to the lower part of the back of the head. The effects of one or more blows were substantial and extensive. The cranial vault and the innermost bones of the skull were broken, with secondary cracks in related areas, reflecting the impact. It is improbable that a heavy weight dropped onto the buried body caused the damage to the skull, mandible and atlas vertebra soon after burial (when bone would still respond as “fresh” tissue), since Individual 1 was at least to some extent beneath the Upper Burial, which showed no such breakage. Another example of possible death from cranial trauma is described in the Supporting Information (S1). An adult male dated to ca. 1400 BP (uncalibrated) was buried to the north of the river mouth, on Milnerton Beach. Contrary to the argument that interpersonal violence occurred only within a brief window of time [27], accumulating evidence suggests that it occurred among South-western Cape forager communities from about 2500 BP onward.

Weapons associated with interpersonal violence in Northern KhoeSan communities are poisoned arrows, spears and clubs [117:388]. Archaeological evidence from south-western Cape is consistent with this list [7]. Could the fragmentary bone point/linkshaft represent an arrow point? Could the Upper Burial have died from a poison arrow? This seems improbable, since the ethnographically known neurotoxins used by KhoeSan hunters take days to kill prey [118].

Does the probable violence associated with the 1997 Unit mean that the entire Lagoon Beach assemblage is violence-related? Not necessarily. Some of the discoveries appear to have been single burials. The evidence of co-interment for the SAM-AP 6041 group is somewhat unclear. While the skeletons were documented to be near one another, no observations were made regarding one or more grave shafts. Taking the presence of children and infants into account, the assemblage may represent a burial ground used by a community for the interment of various members who died of various causes. We cannot know whether violent events like that which may have preceded the creation of the 1997 Unit were common or rare.

### Was this a community burial ground?

A parsimonious interpretation for the discoveries described here is that a forager community identified a section of Lagoon Beach as a burial ground, including expedient burials, and that they had a cultural preference for burying the deceased in adornment and/or garments. By 2000 BP, herding of domestic animals had reached this region [119], but there is no evidence among the biological and cultural information reported here that suggests a herder identity for those buried here. The small sizes of the ostrich eggshell beads are especially informative here. Nearly all the >500 beads measured from Lagoon Beach had external diameters of between 4 and 5 mm. Beads of this size dominate in pre-2000 BP (i.e. hunter-gatherer) contexts in Namibia [120, 121] and in the Western Cape [122, 123]. Small beads persist into the last 2000 years at the site of Witklip, which Smith et al. (1991) interpreted as a hunter-gatherer site, contrasting with the larger ones from the contemporary herder occupations at Kasteelberg A and B. Beads from Kasteelberg A and B are mostly 6 mm or more in diameter [122], a size class that is entirely absent from the Lagoon Beach sample. The beads described here clearly group with pre-2000 BP hunter-gatherer beads. While it has not yet proved possible to distinguish the skeletons of hunters and herders based on biological observations, we note that the dental wear and dietary isotope ratios suggest a diet with a substantial marine component, consistent with values recorded amongst earlier forager societies. Limb strengths are consistent with hunter/forager activities.

Spatially delimited hunter-gatherer burial areas have been documented in the Eastern Cape Province of South Africa [124]. Multiple burials from tightly delimited areas are known from within rock shelters, such as Matjes River Rock Shelter, Whitcher’s Cave and others [40] but these accumulations may be the result of prolonged, intensive occupation at these locales. Instances of burial with many OES beads and grave goods, including painted stones, have been reported in both the Southern and Eastern Cape. It has been suggested that burials with elaborate grave goods from the past 5000 years are indicators of intensified ritual behavior, which was in turn the result of social stress, perhaps related to environmental carrying capacity [125].

Evidence of multiple interments created by Late Holocene south-western Cape foragers has been presented by Manhire at Faraoskop (1993) (as noted earlier) and by Dewar at Saldanha Bay (2010). A possible parallel to the Lagoon Beach burials is that of the “Diaz Street Midden” at Saldanha Bay. Dewar has suggested that the five burials with indistinguishable radiocarbon dates represent people “trying to claim specific locales or territories” (2010:33) within an environment of subsistence intensification and reduced mobility. The attractive environmental feature at Saldanha may have been the rocky shoreline, a source of abundant marine protein. Another point of rocky coastline that has yielded a large number of Late Holocene forager skeletons is Melkbosstrand, although relationships among the >20 dated skeletons from that locale [126] have not been carefully assessed as yet. A factor underlying intensification, presumably, was population growth [127].

## Conclusions

Notable features of the Lagoon Beach burials are their clustering within a small area and short time frame, their diverse individual adornment, and the inclusion of individuals of all ages. There is no clear evidence for social hierarchy (expressed through grave goods); territoriality is possible. The presence of colonial-era artefacts (fragments of clay pipes and Oriental porcelain, noted in site records from 1978) may indicate that this was indeed a focal point on the landscape to which people continued to return even during the colonial period. Nevertheless, as noted by Walker, “It remains a challenge for archaeologists to understand how mobile hunter-gatherers imposed meaning on the landscapes they occupied (2019:146).”

Taking special note of one multi-person interment that was removed *en bloc*, the burials provide a unique window into LSA adornment/garments. They are unique for the region in terms of the quantities of beads, the preservation of sections of beadwork, the inclusion of utilitarian objects and the presence of infants, possibly some with adornment if the perforated shells were worn as beads. We have noted the association of headbands and OES pelvic adornment with men at this site, perhaps reflecting gender identity. Evidence that one group of four people was buried in haste, with one of the four having sustained fatal cranial trauma, provides new evidence for interpersonal violence among foragers of this region in the late Holocene.

Extension to the entire assemblage of the assumption of burial after violent death is not warranted. The demographic range of the assemblage, combined with the absence of evidence for perimortem bone damage, suggests that at least some–and perhaps most–of the people buried on Milnerton Beach died natural deaths and were buried at a chosen spot. Other lines of evidence in support of this being a burial ground come from documents filed at the times of discovery. Some skeletons were documented to have been incomplete, as though they had been disturbed by subsequent interments. Other skeletons were surmised to have been placed in typical flexed postures.

The hypothesis that community burial spaces were a feature of perimeter defense among Late Holocene hunter-gatherers is supported and expanded by the evidence from Lagoon Beach. Not only was this small area used for burying the deceased during a delimited time span, but also there is evidence of distinctive funerary practices: interment of infants in the same area as adults, and interment of the deceased in some garb. We have reported here on these characteristics in two multiple interments: the SAM-AP 6041 group and the 1997 Unit. Such cultural preferences may have distinguished the people who lived around what is now Lagoon Beach from people living elsewhere along the south-western Cape coast. The locale is a long, sandy beach. Areas such as this are not usually a focus of archaeological evidence for hunter-gatherer activities; shell middens tend to be concentrated near rocky shores. Lagoon Beach is adjacent to a lagoon that may have been a particularly reliable source of estuarine/lagoonal foods, like mullet, and non-food resources such as the estuarine grass *Zostera*, which was used for bedding.

The 1997 Unit, in which one skeleton shows evidence of death from blunt force trauma, is consistent with the hypothesis that resource intensification sometimes led to interpersonal violence. While it is possible that this was a conflict between hunters and herders, there is no evidence for or against this. The violence reflected by the 1997 unit is consistent with a pattern that was underway hundreds of years prior to the introduction of domesticated stock [summarized in 27]. The evidence presented here, in combination with a growing body of information for the region as a whole, supports the hypothesis that this was a contested landscape, in which competition and conflict formed part of daily life.

## Supporting information

S1 File Other curated human remains associated with the Milnerton locale. (DOCX)

S1 Table Known human skeletal materials catalogued as coming from “Milnerton”. AOI = area of interest, the patch of beach ca. 200 m south of Milnerton lagoon. The lighthouse is 1.1 km north of the mouth of the lagoon.
(DOCX)

S1 Fig A and B: SAM-AP 6334 cranium viewed from right and left sides illustrating damage to the bone, some of which may be associated with cause of death.
(TIF)

S2 Fig CT scan of SAM-AP 6334 cranium illustrating a depression injury with inward bending, consistent with damage to fresh bone tissue. (JPG)
