# Supplementary material for: A Late Holocene community burial area: Evidence of diverse mortuary practices in the Western Cape, South Africa
Source: PLoS One. 2020 Apr 16;15(4):e0230391. doi: 10.1371/journal.pone.0230391 (PMC7161951; doi:10.1371/journal.pone.0230391)
Supplement: S1 File — (DOCX) [file pone.0230391.s001.docx]

S1 Supporting information: Other curated human remains associated with the Milnerton locale.

SAM-AP 6064: Found 2 km north of the lighthouse, the unit includes the partial skull and relatively complete post-cranial skeleton of a middle-aged adult man, possibly European. The mandible has a prominent chin. The femur is about 450 mm long. The bone looks somewhat fresh and there is some desiccated soft tissue at muscle attachment sites; it may date from the past 100 years. Muscle markings seem quite prominent.

SAM-AP 6236, 6237, 6238: These three units are associated with an accidental discovery during trenching/pipelaying in 1987. All are partial and are assumed to be associated with the date from recent times that was generated from the skull in unit SAM-AP 6238 (published here with the permission of D. Stynder). They came from “Milnerton Ext 13, between Pence Road and Rietvlei” via the Milnerton police.

SAM-AP 6334: We document this skeleton in some detail, as it may be relevant to the developing topic of interpersonal violence in the latter part of the Later Stone Age. The relatively complete skeleton, with bone tissue in excellent condition (although there is breakage and some damage to most bones), is listed in the Iziko record as having come from “Milnerton,” accessioned in 2002. Notes with the bones refer to it as having come in a black crate. No other contextual details are available. One fibula was used to produce a radiocarbon date (1400 B.P., see Table) [2]. When that date was published, and in subsequent publications, the skeleton has been listed as coming from Melkbosstrand [4-6], a stretch of West Coast beach about 25 km north of Milnerton. Although the Iziko catalogue indicates that it came from Milnerton, this skeleton is not likely to have come from the focal region on Milnerton Beach. By 2002, that region had been developed as multi-unit housing. Also, the brownish surface color of the bones suggests that the body was buried in a different, more organic substrate. Its date is at least 300 years more recent than the dates of the skeletons from the focal region.

Extant bones include most of the skull, the mandible, some vertebrae (C2, 5 thoracic, lumbar 1, 2, 4 and 5); sacrum, both hips, ribs in good condition, left scapula missing most of its blade, left clavicle, both humeri, both ulnae, both radii (left radius broken in half, post mortem); both femora, both tibiae, the left fibula, one metacarpal and a proximal manual phalanx, both calcanei, the left talus, right metatarsals, plus one left metatarsal. Other bones (patellae, sternum, hand bones, etc.) are missing.

The skeleton is that of an adult male, based on pelvic indicators. Age-at-death is more than 25 years (all late-fusing epiphyses are closed) but not much older based on a pubic symphysis at Suchey-Brooks [7] phase 2 and a youthful adult auricular surface. The age estimate from rib bone histology (undetermined ethnicity equation [8]) is 32.4 (+/-9) years. Femur maximum length is 409.5 mm, femoral head diameter is 38.6 mm. Hence, his stature was similar to that of other LSA foragers. Bi-iliac breadth is estimated at 225 mm, also consistent with this group [4].

Stable isotope ratios from bone collagen are: δ^13^C -12.1‰ and δ^15^N 14.0‰. The C/N ratio is 3.2, with 43%C and 15.5%N. These values are consistent with a marine-protein dietary regimen. Cross-sectional geometric property of one femur is available. Body size standardized value of *J* at midshaft is 308 (body-size-standardized, as explained in main text), making it somewhat less robust than typical males from this population (see Tables 6 and 7 in the main text).

There are faint markings of ochre on the left side of the vault and mandibular body, suggesting that he may have been lain on his right side and the head subsequently sprinkled with ochre. There are scrapes, striations and a penetrating cut paralleling the left side of the sagittal suture, about 22 mm long, as well as damage to the left nasal margin and the left coronoid process of the mandible (S1 Fig 1A and B). Based on different coloration and the absence of evidence of bone plasticity, this damage appears to be post-mortem. It may be associated with the discovery of the skeleton. However, a CT image of the skull has been assessed as showing a perimortem depression injury on the left side (S1 Fig 2).

S1 Fig A and B. SAM-AP 6334 cranium viewed from right and left sides illustrating damage to the bone, some of which may be associated with cause of death.

S1 Fig 2. CT scan of SAM-AP 6334 cranium illustrating a depression injury with inward bending, consistent with damage to fresh bone tissue.

There is also damage to the right side of the vault in the parietal-temporal region. A crescent shaped piece of parietal (along the squamosal suture) is extant. The release edge is smooth, beveled inward, with no radiating cracks. Such inward beveling is characteristic of trauma to fresh bone. This rounded break is mirrored by a larger, rounded break to the squamous portion of temporal that extends down to the root of the zygoma, producing an oval hole (40 mm wide, maximum of 53 mm longest axis). The squamosal portion of the temporal bone that broke away is not present.

All the exposed, broken bone edges are a yellowish tan color, in contrast to the reddish brown of the skull’s surface. The bevelling and the coloration provide conflicting evidence with regard to evaluating whether this is peri- or post-mortem damage. A conclusion of death through interpersonal trauma must remain tentative, given the absence of evidence for defense wounds on the post-cranial skeleton and the absence of information about the conditions relating to the skeleton’s context at the time of discovery.

**References**

1. Abrahams G. Report on human skeletal remains and associated artefacts from Milnerton Beach, Cape Province. South Afrian Archaeological Bulletin. 1983;38(137):33-5.

2. Stynder DD. A quantitative assessment of variation in Holocene Khoesan crania from South Africa's western, south-western, southern and south-eastern coasts and coastal forelands. Cape Town: University of Cape Town; 2006.

3. Morris AG. A Master Catalogue: Holocene human skeletons from South Africa. Johannesburg, South Africa: Witwatersrand University Press; 1992 1992. 1-157 p.

4. Kurki HK, Stynder DD, Pfeiffer S. Allometry of head and body size in Holocene foragers of the South African Cape. American Journal of Physical Anthropology. 2012;147:462-71.

5. Stynder DD, Ackermann RR, Sealy JC. Craniofacial variation and population continuity during the South African Holocene. American Journal of Physical Anthropology. 2007;134:489-500.

6. Pfeiffer S. Population dynamics in the Southern African Holocene: Human Burials from the West Coast. In: Jerardino A, Braun D, Malan A, editors. The Archaeology of the West Coast of South Africa: Cambridge Monographs in African Archaeology. BAR International Series 2526. 84. Oxford: Archaeopress; 2013. p. 143-54.

7. Brooks S, Suchey JM. Skeletal age determination based on the os pubis: a comparison of the Acsádi-Nemeskéri and Suchey-Brooks methods. Human Evolution. 1990;5(3):227-38.

8. Cho H, Stout SD, Madsen RW, Streeter MA. Population-specific histological age-estimating method: A model for known African-American and European-American skeletal remains. Journal of Forensic Sciences. 2002;47(1):12-8.
