# Supplementary material for: A Late Holocene community burial area: Evidence of diverse mortuary practices in the Western Cape, South Africa
Source: PLoS One. 2020 Apr 16;15(4):e0230391. doi: 10.1371/journal.pone.0230391 (PMC7161951; doi:10.1371/journal.pone.0230391)
Supplement: S1 Table — AOI = area of interest, the patch of beach ca. 200 m south of Milnerton lagoon. The lighthouse is 1.1 km north of the mouth of the lagoon. (DOCX) [file pone.0230391.s002.docx]

S1 Table. Known human skeletal materials catalogued as coming from “Milnerton.” AOI = area of interest, the patch of beach ca. 200 m south of Milnerton lagoon. The lighthouse is 1.1 km north of the mouth of the lagoon.

| **Catalogue** | **Age & Sex** | **Lab ref for date** | **Date BP (uncalib)** | **Notes** | **Citation** |
| --- | --- | --- | --- | --- | --- |
| UCT 321 | “robust” adult male |  | historic | Found near lighthouse | [1] |
| SAM-AP 5032 | Adult male  (cranium only) | OxA-V-2056-35 | 765+/-25 | North of AOI, near wreck of Winton | [2] |
| SAM-AP 6057 | ‘part of skull’ |  |  | North of AOI: Rietvlei | [3] |
| SAM-AP 6064 | Large adult male |  | Probably historic | Found near lighthouse | See notes below |
| SAM-AP 6236 |  |  |  | North of AOI | See notes below |
| SAM-AP 6237 |  |  |  | North of AOI | See notes below |
| SAM-AP 6238 | Young adult male | GrA-9075 | 170+/-50 | North of AOI | See notes below |
| SAM-AP 6334 | Young adult male | Pta-8790 | 1400+/-50 | Locale uncertain | See notes below |
