# Supplementary figures and images for: A Late Holocene community burial area: Evidence of diverse mortuary practices in the Western Cape, South Africa
Source: PLoS One. 2020 Apr 16;15(4):e0230391. doi: 10.1371/journal.pone.0230391 (PMC7161951; doi:10.1371/journal.pone.0230391)

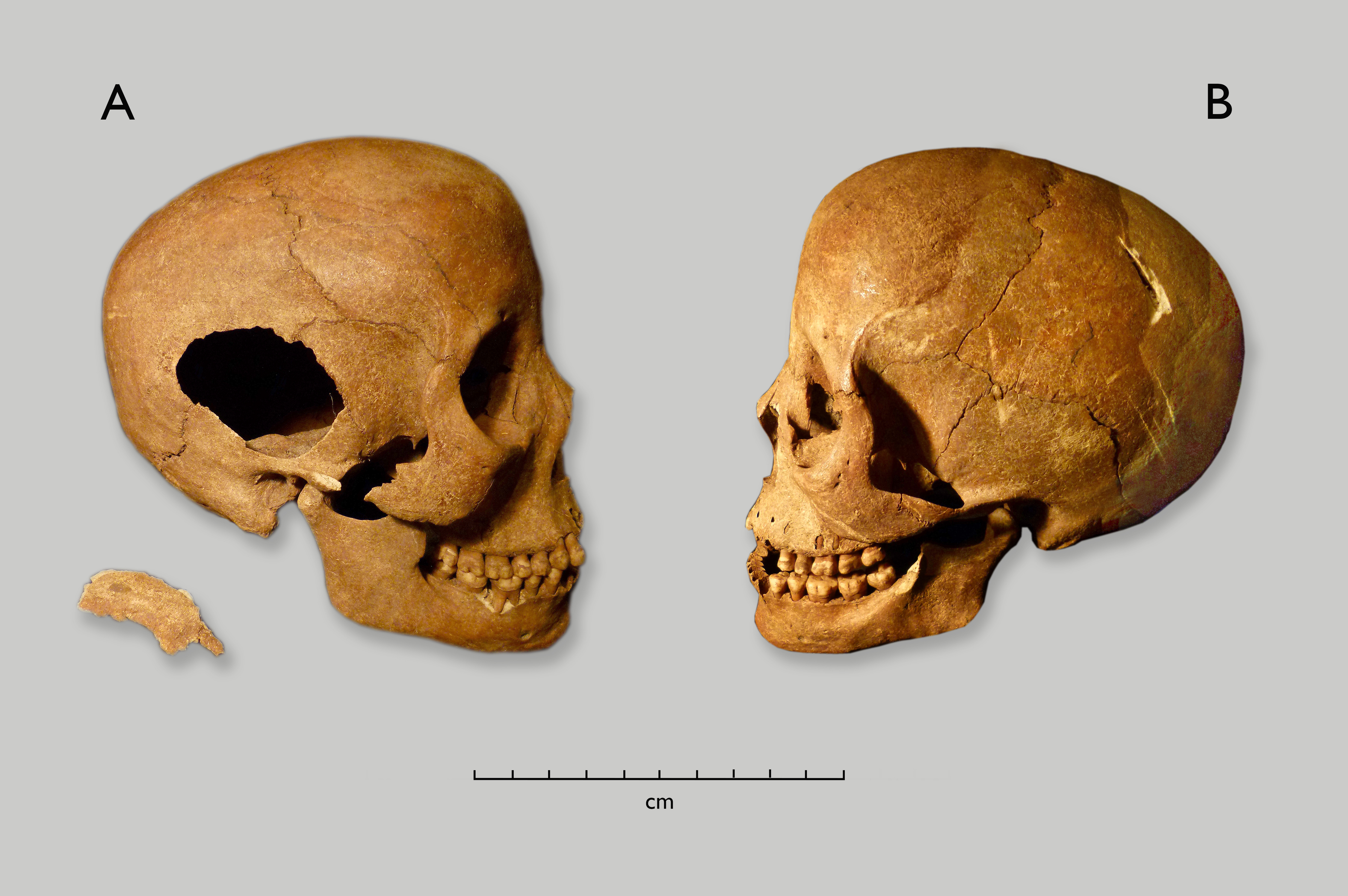

Supplement: S1 Fig — A and B: SAM-AP 6334 cranium viewed from right and left sides illustrating damage to the bone, some of which may be associated with cause of death. (TIF) [file pone.0230391.s003.tif]

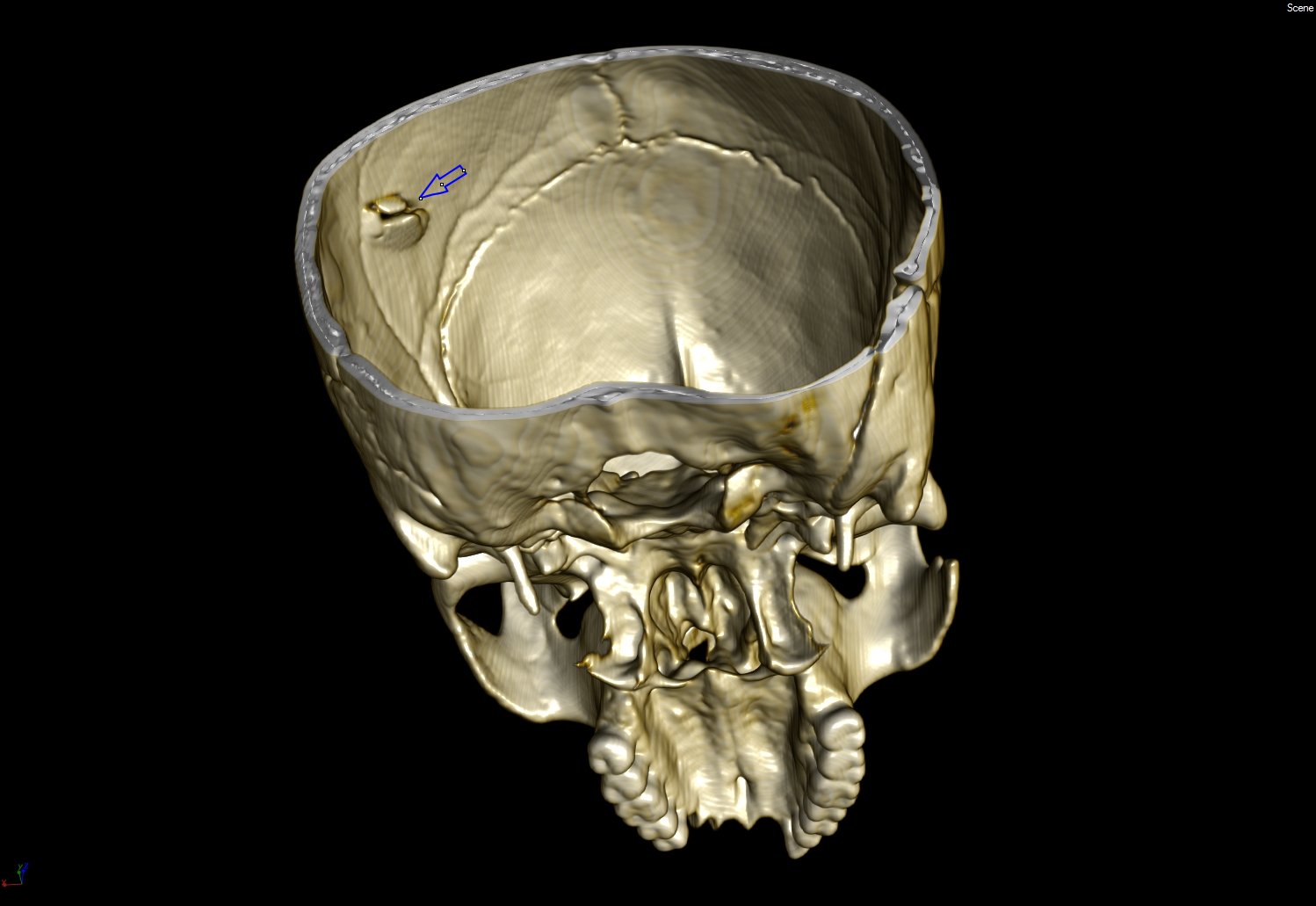

Supplement: S2 Fig — (JPG) [file pone.0230391.s004.jpg]
